# Precision micro-milling process: state of the art

**DOI:** 10.1007/s40436-020-00323-0

**Published:** 2020-10-27

**Authors:** Lorcan O’Toole, Cheng-Wei Kang, Feng-Zhou Fang

**Affiliations:** 1 Center of Micro/Nano Manufacturing Technology (MNMT-Dublin), University College Dublin, Dublin 4, Ireland; 2 State Key Laboratory of Precision Measuring Technology and Instruments, Center of Micro/Nano Manufacturing Technology (MNMT), Tianjin University, Tianjin, 300072 People’s Republic of China

**Keywords:** Precision machining, Micro-milling, Size effect, Deflection, Runout, Tool wear

## Abstract

Micro-milling is a precision manufacturing process with broad applications across the biomedical, electronics, aerospace, and aeronautical industries owing to its versatility, capability, economy, and efficiency in a wide range of materials. In particular, the micro-milling process is highly suitable for very precise and accurate machining of mold prototypes with high aspect ratios in the microdomain, as well as for rapid micro-texturing and micro-patterning, which will have great importance in the near future in bio-implant manufacturing. This is particularly true for machining of typical difficult-to-machine materials commonly found in both the mold and orthopedic implant industries. However, inherent physical process constraints of machining arise as macro-milling is scaled down to the microdomain. This leads to some physical phenomena during micro-milling such as chip formation, size effect, and process instabilities. These dynamic physical process phenomena are introduced and discussed in detail. It is important to remember that these phenomena have multifactor effects during micro-milling, which must be taken into consideration to maximize the performance of the process. The most recent research on the micro-milling process inputs is discussed in detail from a process output perspective to determine how the process as a whole can be improved. Additionally, newly developed processes that combine conventional micro-milling with other technologies, which have great prospects in reducing the issues related to the physical process phenomena, are also introduced. Finally, the major applications of this versatile precision machining process are discussed with important insights into how the application range may be further broadened.

## Introduction

The trend toward miniaturization of precision micro-components, such as for microelectromechanical, nanoelectromechanical, and micro-medical systems, has led to advances in microfabrication techniques in recent years. This demand for micro-sized parts with high aspect ratios has necessitated the biomedical, electronics, automotive, and aerospace industries to adopt and apply both new and old manufacturing processes at the microscale. Although microfabrication techniques have existed for many years, the stringent requirements of extremely tight tolerances on form, dimension, and surface characteristics [1], high machining efficiency, and machine positioning accuracy have led to further developments in precision machining processes [2]. Micro-milling is a precision micromechanical cutting process, which has been developed to facilitate the increasing requirements [3].

Micro-milling is an effective and efficient precision machining process for manufacturing components with microstructures such as complex three-dimensional (3D) surfaces at the microscale. Typically, micro-milling can be characterized by the size of the cutting edge diameter of the micro-milling tool, which lies between the range of 1 µm and 1 000 µm [4], whereas the diameter of the cutting edge in the conventional milling process is greater than 1 000 µm. However, this definition focuses only on the tool and does not incorporate the important aspects of the machining process, namely precision, accuracy, and underlying material removal mechanism. Therefore, a more technical approach to characterize the micro-milling process can be as follows: a precision mechanical cutting process with geometrically defined cutting edge tool diameters of less than 1 000 µm for precise material chip removal to within less than 1 µm tolerance on form and dimensional accuracy [5].

However, the micro-milling process is limited by inherent physical process issues when machining at the microscale, which are not present when milling at the macroscale. Such constraints relate to material removal mechanisms at the microdomain, which include chip formation, size effect, and process stability. Therefore, the major physical processes that limit the process efficiency and precision in micro-milling are thoroughly discussed. The effects of these negative phenomena on the machining process outputs are considered, while insights into how these effects can be minimized, if not eliminated, are presented.

Recently, there has been a strong research interest in the micro-milling process, with much work focusing on the process inputs, such as workpiece material and microstructure, geometry and materials of tools, efficient toolpath generation, and cutting fluid. Examining the latest works concerning the influence of these process inputs on the cutting force, surface roughness, and tool wear during machining provides a clear depiction of the current micro-milling process. This review therefore investigates the theoretical, analytical, and experimental works most recently published to identify areas of the process for future development.

In terms of process advancement, one of the key areas will be in supplementing the micro-milling process with other successful technologies. Section 4 of this review will examine the recent successful studies that implemented secondary systems to produce advanced processes, such as ultrasonic-assisted, laser-induced oxidation-assisted, and plasma jet-assisted micro-milling processes. The importance of such assisted processes will become even more apparent with future developments of higher hardness and wear resistant materials. Such materials are classified as “difficult-to-machine” materials, which include hard and wear resistant superalloys, refractory metals, structural ceramics, composites, polymers, and magnesium alloys [6]. The consideration is that the limitations of the micro-milling process in terms of physical process constraints, i.e., chip formation, size effect, and process stability, may not be overcome by micro-milling alone in the future.

The application of micro-milling across the biomedical, electronics, automotive, and aerospace industries is also discussed in relation to the versatile nature of the precision machining process. Such application ranges from machining of microstructures and textures to precision machining of very hard and wear resistant materials for utilization in the mold manufacturing industry. Therefore, this review introduces the current state-of-the-art micro-milling process, beginning with the issues of physical process phenomena associated with machining at the microscale and including an in-depth examination of how to minimize these negative issues. The process inputs are examined in relation to the process outputs, offering insights into areas for improvement in the future, which will further advance the development of the micro-milling technology. Finally, applications of the micro-milling process are described in detail, followed by insights into the future perspective of micro-milling. The objective of this work is to present the development, benefits, applications, limitations, and future insights of micro-milling and to discuss the recent publications regarding this precision machining process.

## Fundamentals of the process

Because the precision micro-milling process has been developed from conventional milling, the two milling processes share many similar characteristics such as machine components and configuration, tool geometry, and cutting fluid. However, the material removal mechanisms between these two mechanical cutting processes cannot be mutually correlated. Conventional milling primarily considers shearing forces acting on the rake face and far lesser ploughing forces on the flank face [7], which are mainly caused by machine chatter stability [8]. The shearing-dominant regime is the desired material removal mechanism during any cutting process, where material is removed as distinctive chips along the rake face. The ploughing-dominant regime is the unwanted material removal mechanism, where material deforms plastically under the flank face and no chips are formed. The ploughing-dominant regime results in extremely poor surface finish and very high tool wear due to high cutting and friction forces, high temperature, etc., during machining. In contrast to precision milling, micro-milling is subject to both considerable ploughing and shearing regimes. The contribution of each mechanism depends heavily on numerous factors, such as chip formation, undeformed chip thickness (UCT), size effect, tool deflections, and process stability. Each of these factors can also significantly influence each other, leading to a more dynamic and complicated effect when determining which material removal mechanism will dominate during micro-milling.

### Chip formation

**Fig. 1:**
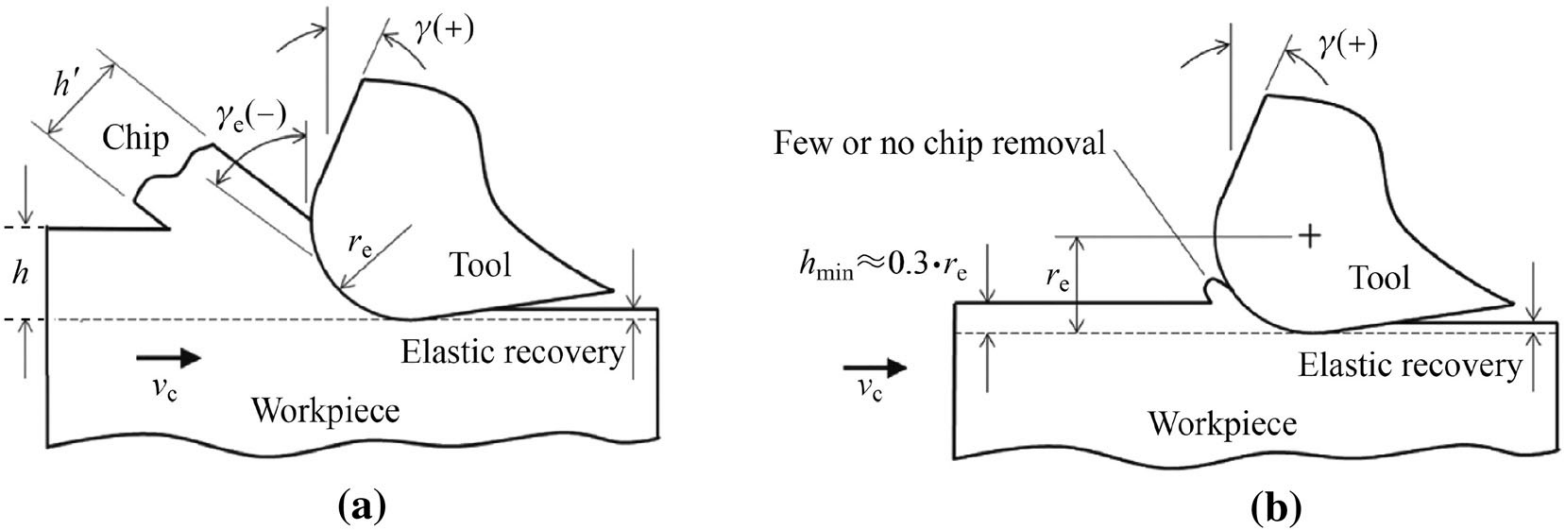
Chip formation mechanism of **a** macro-milling and **b** micro-milling in terms of minimum undeformed chip thickness *h*_min_ and cutting edge radius *r*_e_ (Adapted and reprinted from “Machining scale: workpiece grain size and surface integrity in micro end milling” by Rodrigues and Jasinevicius [28], with permission from Elsevier)

The minimum chip thickness is the critical limit determining whether the material flows along the rake face, forming chips as the shearing mode of material removal, or along the flank face causing elastic or plastic deformation depending on the material, as the ploughing mode [9–11]. Therefore, it can be defined simply as the minimum UCT, below which a defined chip cannot be formed stably. This critical value will depend on the process parameters, material properties, and microstructure [12]. When the UCT is less than the minimum value, chips due to the ploughing-dominant mode of material removal will not be generated. In contrast, when the UCT is larger than the minimum value, a defined chip will be generated and the process can be compared to that of conventional milling [9] (see Fig. 1). Consequently, there will be no chip removal at very small depths of cut during micro-milling. Instead, the workpiece will undergo pure elastic deformation when the cutting tool passes through the workpiece material, which then recovers to the original height. However, with an increase in the depth of cut, the material instead begins to plastically deform. With the continuous increase in the depth of cut, the material removal mechanism then begins to shift from plastic deformation to shear chip formation, if the minimum UCT approaches a certain threshold. Therefore, chips can be formed and removed only when the depth of cut exceeds the minimum UCT [13].

The UCT is one of the most important aspects that determine which material removal process will dominate in micro-milling, and it can be influenced by many factors [14]. The combined effects of factors such as tool setting errors and toolholder and spindle errors will result in significant tool runout of the cutting edge with respect to the workpiece [15]. However, the minimum UCT will be mainly affected by the tool geometry and material [16], workpiece material, and microstructure [17]. Generally, the UCT is developed as a prediction model because it is not necessarily a physical parameter and cannot be identified directly during machining. The results of measurements such as cutting force and surface integrity can be examined to determine which mechanism of material removal is dominant during machining. Up until recently, the mechanical models for micro-milling were based on scaling conventional milling models with adaptations [18, 19] and investigation of single factor influences. As discussed however, simply reducing the scale from macro to micro does not present a constitutive model. More importantly, micro-milling is far more complex owing to numerous factors having significant influences on the process. The models for UCT in micro-milling are established to predict process outputs, such as cutting forces, surface quality, and temperature, as well as to predict fundamental physical processes, including the process stability and material removal mechanisms. Therefore, to select optimal machining parameters, the material removal behaviour during micro-milling operations must be fully understood and implemented by accurate models. The establishment of such UCT models in micro-milling is clearly an important research topic to obtain a much higher precision and more efficient micro-milling process.

The early work by Son et al. [20] on ultra-precision diamond cutting found that the minimum UCT was determined by the tool edge radius and the friction coefficient of the workpiece-tool interface. Their work then led to important research by Malekian et al. [21], who confirmed that the minimum UCT was a function of both the edge radius and friction coefficient and was dependent on the tool geometry and properties of the workpiece material. Through their proposed analytical model based on the minimum energy principle and infinite shear strain method, the normalized minimum UCT of Al6061 was approximated as 0.23 of the edge radius. However, it was noted by the authors that the minimum UCT was a range of values, rather than a single point. This may be attributed to the stagnation region, instead of a stagnation point, as observed by others. Ramos et al. [22] also developed a model for estimating the minimum UCT of AISI 1045 based on their experimental results. The minimum UCT was found to substantially decrease with higher cutting velocities and to moderately increase with higher cutting edge radii. Such prediction models that estimate the minimum UCT are important because they can help to minimize the amount of ploughing-dominant material removal and offer the optimum cutting conditions. One example of the importance of prediction models is when working with materials such as magnesium, where the risk of fire is a major concern during high-speed cutting, because magnesium in the molten state is flammable when exposed to oxygen. Therefore, accurate models to predict cutting temperature at the flank face in relation to the UCT are very important, as determined by Fang et al. [23].

Chen at al. [11] developed a model of chip formation, which was capable of connecting the minimum UCT, UCT, and periodicity of cutting force together. Their model can predict the normalized value of minimum UCT (*λ*_e_), which represents the ratio of the minimum UCT to the cutting edge radius *r*_e_. They estimated this value to be 0.43 ≤ *λ*_e_ ≤ 0.48 for cutting edge radii between 2 µm and 3 µm for potassium dihydrogen phosphate crystal, which was another difficult-to-machine material owing to its properties of being soft, brittle and deliquescent. Their systematic work could serve as a reference for similar works on other difficult-to-machine materials, and obtained results could potentially guide the selection of cutting parameters and cutting edge radii for improving the integrity and quality of machined surfaces in the micro-milling of other brittle materials.

Recently, Lu et al. [24] investigated the tool trajectory in micro-milling, with the aim of building a more accurate UCT prediction model while taking into consideration radial tool runout on the cutting edge as well as determining the effect of tool setting errors on the UCT. Comparisons of cutting forces under this UCT model with experimental data indicated that their model could be used to accurately predict cutting forces during the micro-milling process, offering theoretical insights into micro-milling force models for further study. The construction of such accurate, instantaneous undeformed cutting thickness models is important to further establish cutting force models.

The transition of material removal mechanism from shearing to ploughing is an important phenomenon when machining at the microscale [25, 26], as can be seen from Fig. 1. The ductile mode of material removal dominates when the UCT decreases to sufficiently small values during micro-milling, particularly at the submicron level as described above [27]. However, the transition between the shearing-ploughing modes of material removal remains a large issue during the micro-milling process, while such factors as the minimum UCT, size effect, effective rake angle, and tool edge radius, all influence the process of chip formation, leading to one mode of material removal into another. Therefore, a quantitative identification of the chip formation process and its influence on other micro-milling phenomena, such as built-up edge (BUE) and burr formation, is a crucial aspect for research to move forward, for all types of materials [13]. The scientific and systematic understanding of the multifactor effect will become even more significant in the future, particularly when dealing with the increasingly stringent requirements for industrial scale applications of the micro-milling process.

### BUE

**Fig. 2:**
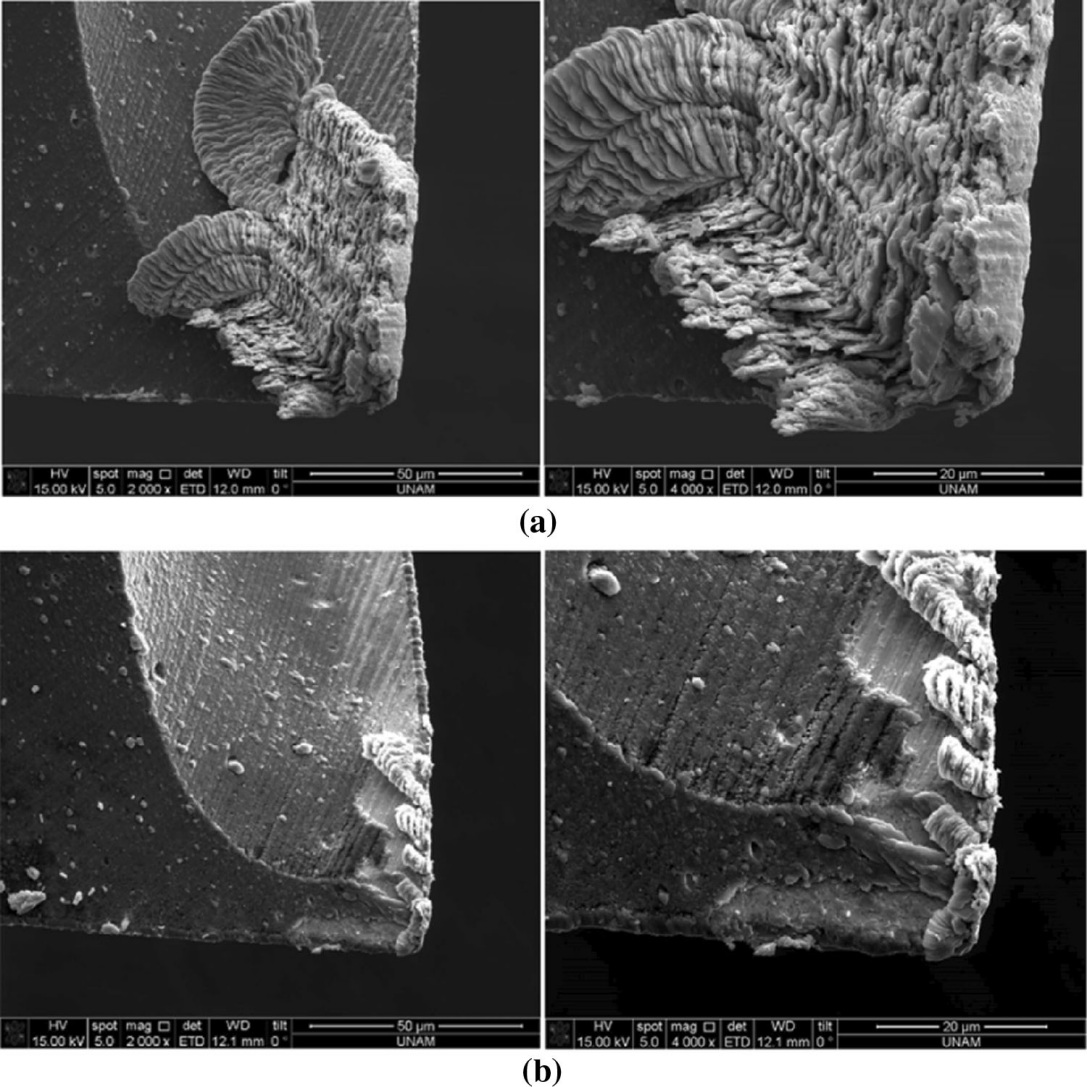
Scanning electron microscopy (SEM) images of both flutes of a micro-milling tool rake face exhibiting uneven BUE on each flute **a** Edge 1 and **b** Edge 2 (Adapted and reprinted from “Microstructure effects on process outputs in microscale milling of heat treated Ti-6Al-4V titanium alloys” by Ahmadi et al. [39], with permission from Elsevier)

When ductile materials, such as aluminum, steel, and even some titanium alloys, are machined using the micro-milling process, BUEs can be observed on the rake face of the tool, as illustrated in Fig. 2. This is due to the adhesion of chips or material onto the cutting tool face, which greatly affects the process outputs and has a significant negative effect on surface roughness, also causing such problems as higher cutting forces and shorter tool life [29]. Because the BUE periodically develops and breaks off the tool rake face, the UCT is affected, which further leads to poor surface quality as well as deposits and smeared regions on the machined surface. The BUE is typically more prominent when using lower cutting speeds, such as in conventional milling. However, it remains an important issue in micro-milling, where even small deposits of adhered material on the cutting face will have considerable negative effects.

The BUE formation in metal machining has been a well-known phenomenon, with research into its unwanted effects beginning even before the 1970s [30]. Similarly, much work has been conducted on the BUE in conventional milling more recently, such as Children’ work toward simulating this phenomenon [31] or Ozcatalbas’ study of orthogonal cutting, which indicated that the BUE affected the chip formation and cutting ratio for different cutting conditions [32]. However, the influence of BUE on the micro-milling process has not yet been characterized in detail. Thepsonthi and Özel [33] carried out investigations on 3D finite element (FE) modeling and simulation of the micro end milling process for Ti-6Al-4V to determine the influence of increasing tool edge radius due to wear on the process performance. They found that the BUE might be formed after the tool was severely worn. More recently, Wang et al. [34] presented one of the first experimental investigations on the effects of BUE on surface quality and its prediction in micro-milling. They studied the influence of BUE while machining 316L stainless steel and reported that the BUE was the main cause of surface finish deterioration in micro-milling besides the chip load. They also showed that when the BUE was not present, theoretical surface roughness models yielded acceptable predictions. Davoudinejad et al. [35] confirmed that the presence of BUE generated unequal chip load and chip formation among different tooth engagements. Their results also proved that burr height was negatively affected by the presence of BUE. Finally, analysis of their results confirmed the importance of the developed 3D FE modeling approach for future work.

Ucun et al. [36] and Aslantas et al. [37] both investigated how coated tools could minimize the BUE to improve surface integrity. Ucun et al. [36] confirmed that a diamond-like carbon (DLC) coating could be used in micro-milling of Inconel 718 to substantially reduce the BUE and burr formation, which improved surface roughness. On the other hand, Aslantas et al. [37] showed that DLC, titanium aluminum nitride (TiAlN), and tungsten carbide carbon layer-coated tools showed better performance against BUE formation than nanocrystalline diamond-coated and uncoated tools.

The BUE affects the friction conditions at the tool-chip and tool-workpiece interfaces by acting like a cutting edge so that the cutting tool material is no longer in contact with the chip and the machined surface. This suggests that a stable BUE formation may protect the tool from rapid wear, leading to a higher machining efficiency. Oliaei and Karpat [38] investigated the relationship between stable BUE formation and process outputs in the micro-milling of Ti-6Al-4V using an experimental approach, taking into consideration tool geometry, surface roughness, and process forces. Their results determined that it was possible to customize a micro-milling tool to have stable BUE formation and design it to machine titanium alloys with long tool life and acceptable surface quality. They concluded that a micro end mill with a low clearance angle yielded the most stable condition for BUE formation, while a large unstable BUE would result in surface quality deterioration. Therefore, the ability to predict and control the BUE size, together with a customized tool design, may be beneficial in the micro-milling of other difficult-to-machine materials. Clearly, simulation models and prediction models that will quantify the dynamic mechanisms of BUE formation, such as tool coating, tool wear, process parameters, and workpiece material properties, are important aspects for future research in micro-milling. Additionally, understanding the chip morphology as well as stable and uniform BUE formation will have significant effects in prolonging tool life, increasing machining efficiency and improving surface quality.

### Burr formation

**Fig. 3:**
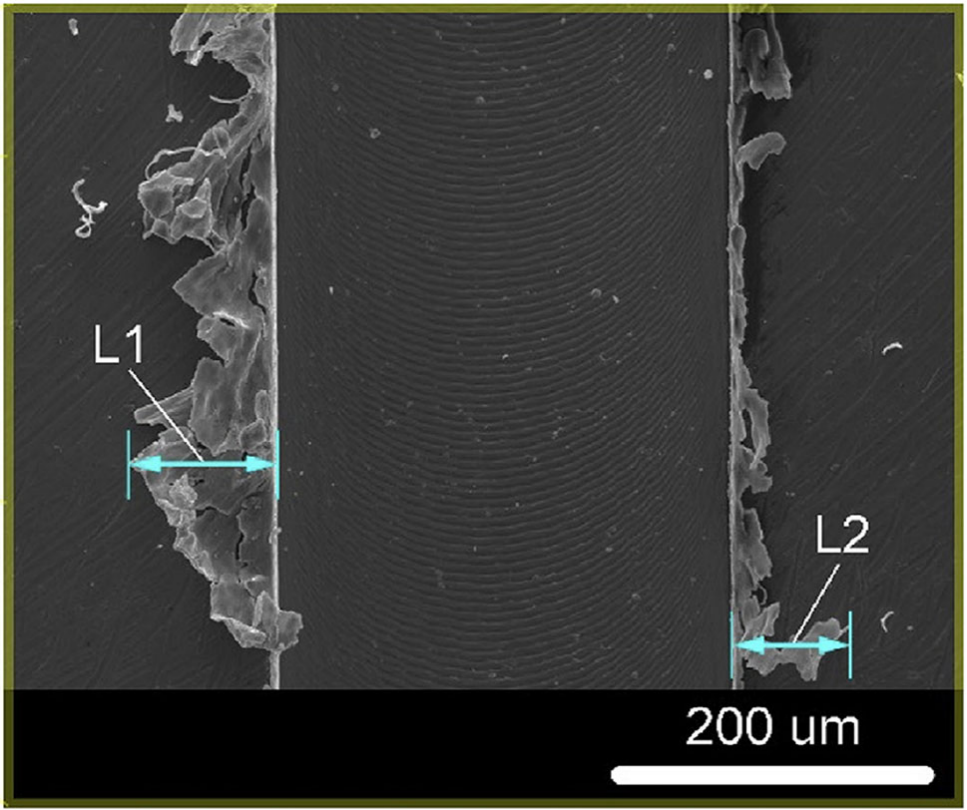
SEM 500× magnification of a machined slot for burr width measurement (Adapted and reprinted from “Novel method for burrs quantitative evaluation in micro-milling” by Medeossi et al. [52], with permission from Elsevier)

**Fig. 4:**
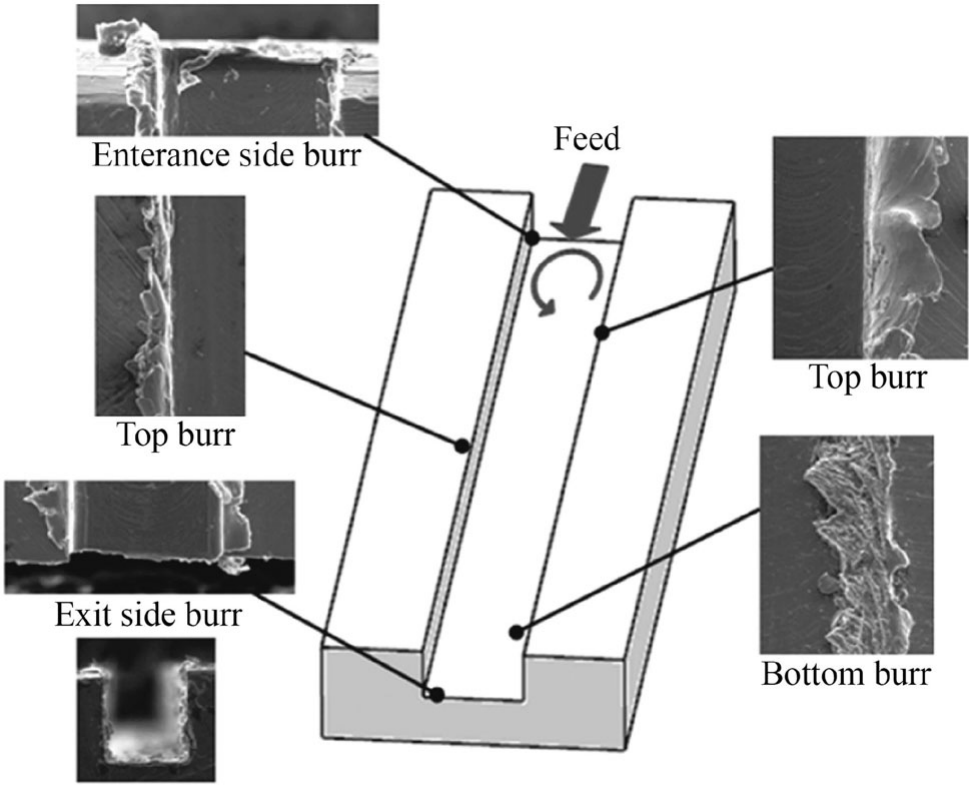
Types of milling burrs (Reprinted from “The effect of spindle speed, feed rate, and machining time to the surface roughness and burr formation of aluminum alloy 1100 in micro-milling operation” by Kiswanto et al. [51], with permission from Elsevier)

A major issue during micro-milling pertains to the formation of burrs, which is an accumulation of material forming a raised edge or volume on the workpiece surface, as can be seen from Figs. 3 and 4. Burr formation is a complicated mechanism involving plastic and elastic deformation, which can be influenced by material properties, tool geometry, and even process instabilities, such as tool runout [40, 41]. It affects the quality of the machined surface significantly, reducing the capability of the part to meet the desired performance and thus the required functionality. The effect is even more significant at the microscale for precise and freeform components; however, burr reduction, characterization, and evaluation remain to be challenging tasks facing the micro-milling process. In addition, burr formation not only decreases the machined part surface and assembly quality, but also increases the production cost by up to 9% of the total machining cost [42]. This is due to a second machining operation, so-called deburring, which may be necessary to remove such materials from machined edges and holes. While the complexity and degree of deburring will depend on a number of factors including burr size, location, and material [43], the focus of research should instead be on the reduction and altogether elimination of burr formation during the micro-milling process through tool geometry development, suitable machine parameters [44], and toolpath optimization. As verified by Fang and Liu [45], although burrs may not be eliminated completely through optimization of the cutting parameters in micro-milling, they may be minimized to less than 25 nm in height. Among the most important factors are UCT and tool sharpness, further showing that an optimal tool geometry is necessary to reduce burr formation [45].

Jin et al. [46] determined early on that the feed per tooth had a major impact on the surface topography in micro-milling and therefore proposed to use higher feed rates. At low ratios of feed per tooth to cutting edge radius, high amounts of burrs are obtained in micro-milling. Saptaji et al. [47] revealed that top burrs could be reduced by either strengthening the side edge of the workpiece or introducing a taper angle in the micro-milling tool. Their results suggest that a combination of a large tool taper and large side edge angle produces the minimum burrs. Although a tapered wall angle, also known as draft angle, is essential in mold machining, it may not always be a desired feature. Chern [48] classified burr formation into five types based on in-plane exit angle: knife-type burr, wave-type burr, curl-type burr, edge breakout burr, and secondary burr. Hashimura et al. [49] classified burrs by location, shape, and formation mechanisms. Litwinski et al. [50] acknowledged bottom burrs in their toolpath planning concept; however, they provided no insights into bottom burr formation or prevention. Kiswanto et al. [51] then performed a significant study concerning top, bottom, entrance, and exit burr formation, as well as the effect of tool wear on burr formation mechanisms, as shown in Fig. 4. Furthermore, the team analyzed the average sizes of top burr for each cutting parameter to determine the relationship between the cutting parameters and burr formation. Their results showed that bottom burr occurred during longer machining times, in comparison to top, entrance, and exit burrs, due to the deterioration of the tool. Therefore, tool wear due to machining time was found to be the most influential factor affecting burr formation. The team also determined that in order to produce a burr-free component, it was recommended to perform up milling during the micro-milling process. Finally, it was shown that appropriate selection of cutting parameters could minimize burr formation. Their important work provided adequate knowledge of appropriate cutting parameter selection during the micro-milling operation of aluminum alloy 1100 to produce a product with minimum burr.

More recently, Medeossi et al. [52] proposed a novel method for quantitatively evaluating burrs based on optical microscopy using an innovative approach to take advantage of the a priori information on the manufacturing operation and an unconventional use of void pixels for rapid and non-destructive evaluation of multiple geometrical quantities. They applied their proposed methodology to slotting micro-milling operations on pure titanium grade II. The results showed that their method had the potential for on-machine monitoring of burr evaluation during micro-milling operations, which had further potential in reducing and eliminating burr formation through process optimization. However, it was noted by the authors that appropriate modeling of the specific machining operation was necessary. Moreover, there are inherent limitations of online vision-based measurement techniques, such as difficulties in measuring burr height or burr features over freeform surfaces without the additional cost of extra rotary axis or right-angle optics for the online measurement system.

In the micro-milling of slots, the relative size of burrs formed on the up-milling side is smaller than that on the down-milling side, as can be seen from Fig. 3. To take advantage of this cutting phenomenon and chip formation mechanism, Chen et al. [53] investigated the effect of vibration assistance in the feed direction during micro-milling of Ti-6Al-4V alloy. By inducing alternating changes in the relative direction of movement between the workpiece and the tool on both sides of the slot through small amplitude, high frequency vibrations, chip formation on both sides of the slot then became similar, leading to less burr formation on both sides of the slot. The results from their FE model simulation and experimental work confirmed the benefit of vibration assistance, which reduced the average top burr height on the down-milling side by 87%. However, this proposed method in burr reduction only utilized vibration assistance in the feed direction and had only been applied for slot micro-milling. Further work is necessary to optimize even this basic unidirectional vibration-assisted micro-milling process. Li et al. [54] provided some of this developmental work also in the feed direction. They determined that larger vibration amplitudes actually increased the exit burr size. Hence, larger vibration frequencies and smaller vibration amplitudes are recommended. Clearly, much more work is necessary to apply vibration in two or three directions for burr removal during freeform surface machining and end milling operations, including both theoretical and experimental works.

Any burr left on the machined surface deteriorates the component quality, precision, function, and performance. This is particularly true for microparts and features. Therefore, burr minimization, and where possible elimination, is essential for high-quality micro-milling operations. This is achievable through extensive research on control techniques and further investigations into understanding the phenomena. Such key areas of interest for future work therefore lie in cutting parameter optimization, toolpath generation, tool geometry and material, and tool coatings and lubrication investigations.

### Size effect

Micro-milling raises significant issues when removing material at the microscale owing to the effect of scaling, otherwise known as size effect. It has been shown that the size effect modifies the mechanism of material removal in conventional milling [55, 56]. However, the characterization and exact cause of this effect remain a point of contention among researchers, indicating that many factors influence chip formation and material removal mechanisms at the microscale. In simple terms, size effect is a phenomenon that modifies the material removal and chip formation mechanisms at the microscale [57]. In conventional milling, the shearing mode of material removal dominates, which leads to chip formation. However, the size effect becomes more significant as the machining scale is reduced to the microlevel, where ploughing of the material surface dominates. This phenomenon produces a major challenge of preventing chip formation by a tooth during a cutting pass, which leads to high cutting forces, high friction, high temperature, and significant tool wear. However, as mentioned earlier, the exact characterization of the size effect has not been fully agreed upon. As an example, Qin [58] defined it as the relationship between the specific energy during cutting and the tool rake angle, which were two important physical parameters that affected the chip removal process. As the depth of cut decreases, the effective rake angle increases, influencing the specific energy. Therefore, the larger the rake angle, the greater the specific energy, which has been widely accepted as the main contributing factor to the size effect phenomenon [59]. Experimental observation by Mian et al. [60] determined that the specific energy, besides the burr root thickness and surface roughness of machined surfaces, could be used as a relevant measure of the size effect in micro-milling. The team also used wavelet transformation to extract energy bands related to the deformation mechanisms involved in machining, while high frequency bandwidths in the acoustic emission signals could also be exploited to identify the size effect phenomenon. The size effect can also be described as the phenomenon whereby the ratio of the UCT to the cutting edge radius of the tool, or the grain size of the workpiece material, will influence chip formation, material removal mechanisms, and material flow, as shown in Fig. 1. This effect can become significant when the thickness of the material to be removed is of the same order of magnitude as the tool edge radius or grain size of the workpiece material [60]. The influence of tool edge radius on the size effect has also been demonstrated through a strain gradient plasticity-based FE model of orthogonal micro-cutting by Liu and Melkote [61].

Actually, the above two definitions are correct because many factors will affect the chip formation and material removal mechanisms; thus, it can be simply said that the size effect is characterized by a nonlinear increase in the energy consumed per unit volume of material removed as the UCT decreases to the same order of magnitude as the cutting tool edge radius or grain size [60, 62]. Therefore, it is very clear that conventional milling mechanisms cannot be used to describe the micro-milling process, because simply reducing the scale of the system will not reproduce the same representative model [63]. The size effect becomes even more significant at the nanoscale, particularly for nanometric cutting, where ploughing of material dominates, rather than shearing and chip formation [64]. Consequently, this variation from the general behavior of both the tool and the workpiece microstructure at the microscale during machining will depend on many factors, such as the material properties and microstructure [39], micro-milling tool parameters [59], machining parameters [65], as well as tool specifications [1, 66]. The physical mechanisms that govern the size effect will be discussed in the following section, including the specific energy, shearing and ploughing-dominant modes of material removal, as well as the effect of tool edge radius.

### Tool edge radius

A small tool edge radius, rather than a sharp point, is an important feature of micro-milling tools to limit crack initiation and failure points at the cutting edge of the tool. However, because of the size effect, downscaling of conventional milling tools makes the cutting edge radius of microtools comparable to the instantaneous UCT. Micro-milling tool edge radii are usually less than 5 μm; however, they can be up to 20 μm [67]. This means that the tool edge radius is in the same order of magnitude as the chip being formed [68], leading to an increase in cutting force [69, 70] and surface roughness [71]. In micro-milling tools, the edge is deliberately rounded to impart strength, prevent plastic deformation, and avoid early tool breakage [72]. Therefore, chip formation occurs along the rounded edge of a tool, resulting in a negative value of the effective rake angle, even if the nominal rake angle is positive [73].

**Fig. 5:**
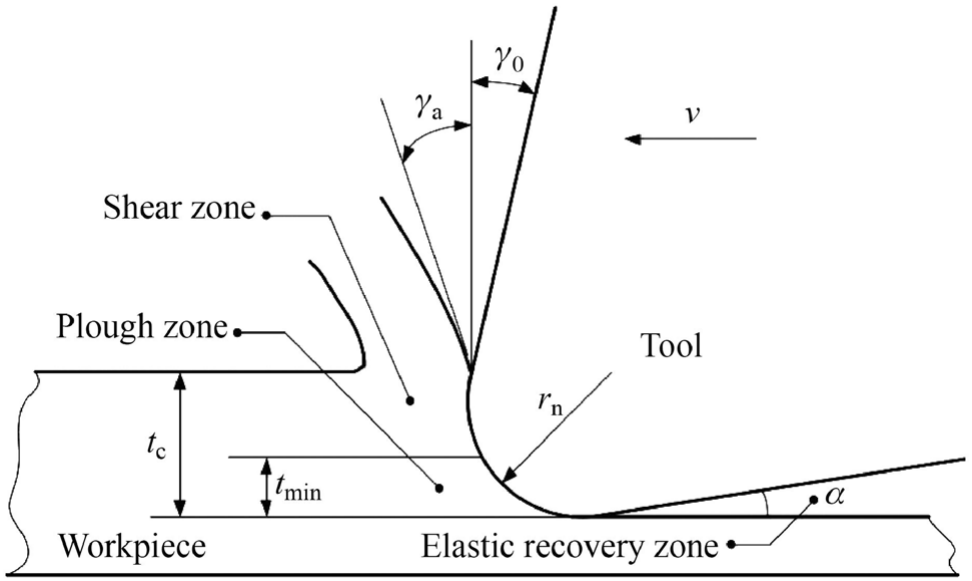
Cutting model of microtool edge showing ploughing, shearing, and elastic recovery zones as a result of tool edge radius *r*_n_ (Reprinted from “Experimental research on micro-milling force of a single-crystal nickel-based superalloy” by Gao and Chen [76], with permission from Springer Nature)

Vipindas et al. [74] presented an investigation on the effect of cutting edge radius on cutting force, coefficient of friction, surface roughness, and chip formation during micro end milling of Ti-6Al-4V, for a wide range of feed per tooth. It was found that the feed per tooth within 1 μm range was the critical value, which was approximately one-third of the cutting edge radius. Below this critical value, the size effect is predominant, leading to the ploughing mode of material removal, as illustrated in Fig. 5. Moges et al. [75] developed a comprehensive mathematical model that incorporated the edge radius of the micro-cutting tool, so that a more accurate prediction of cutting force models could be obtained. Therefore, even though rounding of the cutting edge was necessary in micro-milling tools, an extremely large tool edge radius would greatly influence the size effect. This suggests that a stronger cutting edge to prevent crack initiation could reduce the size effect issue, as it would lead to a smaller radius requirement, which in turn would result in a more dominant shearing mode of material removal. To fulfil this demand, further investigation on the cutting edge tool geometry is necessary.

### Specific energy

**Fig. 6:**
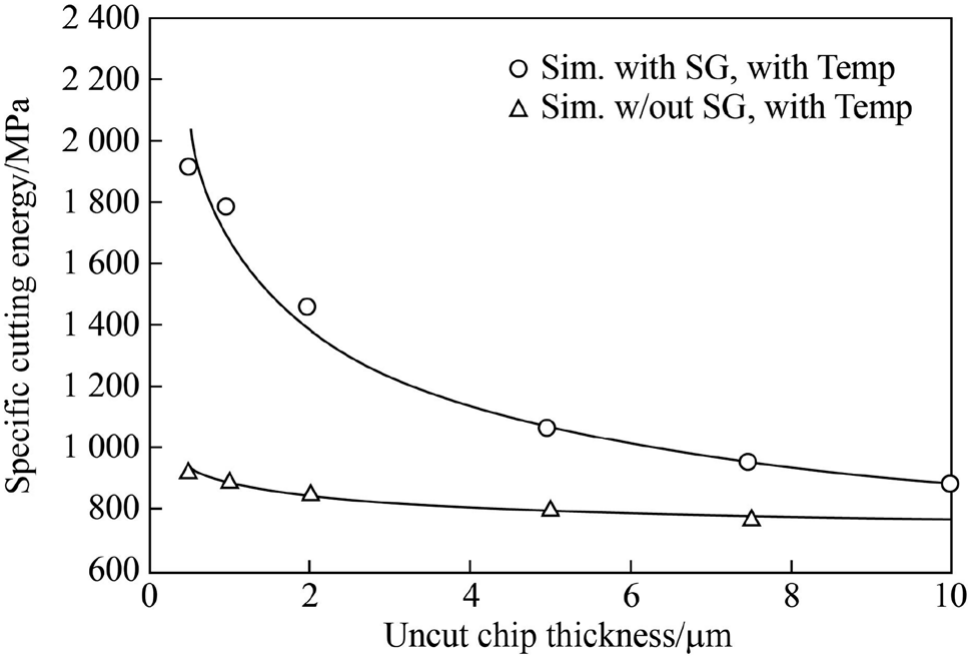
Variation in specific cutting energy with uncut chip thickness at 240 m/min (Adapted and reprinted from “Size effects in manufacturing of metallic components” by Vollertsen et al. [83], with permission from Elsevier)

The energy consumption during the machining process affects both the environmental and manufacturing costs. Therefore, evaluating and limiting the energy consumed during micro-milling can lead to more efficient manufacturing [77]. One such way, according to Fang et al. [78], is to compare the experimental cutting force and specific cutting energy. To compare the energy consumption during machining operations such as micro-milling, the specific energy parameter, which was defined by Li and Kara [79] as the energy consumed to remove a unit volume of material, may be used [80]. The specific energy is a particularly important parameter to consider during micro-milling, as it can be used to evaluate the cutting effectiveness of the process. The ratio of specific energy to the UCT can be helpful in characterizing the size effect in relation to surface generation, as can be seen from Fig. 6.

It has been shown that the size effect strongly affects the specific energy necessary for material removal through chip formation mechanisms, which can alter the material removal mechanisms [16]. An experimental investigation was carried out by Yao et al. [81] to determine the relationships between the specific cutting energy, chip morphology, and surface integrity of martensitic aged steel. They proposed a new method for calculating the effective energy and non-effective energy by the criterion of whether it contributed to chip formation or not, respectively. Their results showed that chips became more segmented with decreasing proportion of the effective energy, whereas increasing the proportion of the non-effective energy resulted in surface integrity deterioration and contributed to the formation of a plastic deformation layer. Then, by assessing the trade-off between surface quality and specific cutting energy, optimized machining parameters were suggested to achieve a precision surface finish with low specific cutting energy and high energy efficiency, which had significant application for the realization of sustainable manufacturing. Gao et al. [59] examined the size effect in relation to the tool edge radius and cutting parameters on specific energy in micro-milling of heat resistant stainless steel. They showed that the specific cutting energy could be fully controlled by regulating the geometrical characteristics of the cutting tool, i.e., the cutting edge radius, and by the machining parameters recommended by their developed minimum chip thickness prediction model. Precise control of the specific energy during micro-milling can therefore lead to more efficient chip formation, which has great significance on improving machining efficiency, tool life, and surface quality. Lauro et al. [82] also analyzed the influence of the size effect on the specific cutting energy of AISI H13 steel in relation to austenitic grain size, while examining the response from a cutting force perspective. They observed that the grain size had a significant influence on both cutting force and specific cutting energy in micro-milling. Their results revealed that larger grain sizes displayed lower specific energy compared with smaller grain sizes. They also showed that increasing the feed rate had a significant effect on reducing specific energy (approximately by 70%) for both small and large grain sizes. Therefore, the recent research suggests that by reducing the specific energy during cutting, the size effect was therefore lessened, resulting in improved machining efficiency, tool life, surface finish, and material removal rates.

### Process stability

Relatively large form error and poor component geometric accuracy are still major obstacles toward achieving higher precision in the field of micro-milling. The main cause of these inaccuracies is the inherent process instabilities during the micro-milling process. Among several factors, the influences of tool deflection, tool runout, and machining chatter are the main sources of surface and dimensional accuracy errors in micro-milled components. These process instabilities further lead to high cutting forces, excessive tool wear, and tool failure, as well as high cutting temperatures, as a result of frictional forces due to rubbing and ploughing during unstable machining conditions, as will be discussed in this section. Because of the relatively low strength and stiffness and very small cutting diameter of micro-milling tools, micro-milling must be performed at very high speeds between 20 000–100 000+ RPM, to ensure productive machining. Moreover, material removal rates can be maintained during the process by increasing the spindle speed to negate the effect of the small cutting diameter of the microtool and relatively slow feed rate. However, high-quality precision air bearing spindles with closed loop position and very accurate speed control are necessary for high RPM machining to ensure process stability. Furthermore, vibration and instabilities during high-speed micro-milling must be minimized, whereas feed rate and positioning must be smooth and continuous [84]. Therefore, it is necessary to develop accurate and reliable process stability models to analyze and improve the performance of such processes as tool runout and tool deflection, as well as minimize self-excited vibration, also known as chatter.

### Tool deflection

**Fig. 7:**
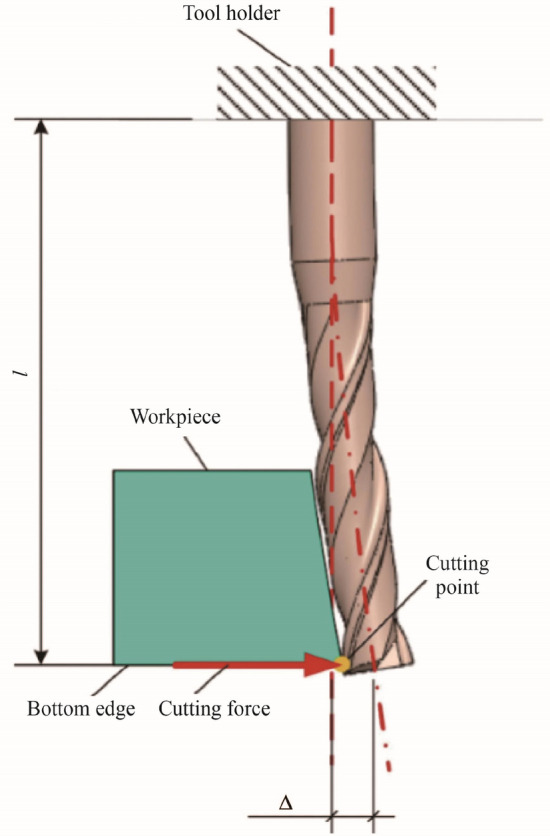
Deflection of milling tool at the bottom of the workpiece edge (Adapted and reprinted from “Analysis of tool deflection errors in precision CNC end milling of aerospace aluminum 6061-T6 alloy” by Nghiep et al. [95], with permission from Elsevier)

Tool deflection is one of the most significant factors limiting the performance of micro-milling processes, particularly limiting form accuracy and precision [85, 86], as can be observed from Fig. 7. A micro-milling cutting tool is severely prone to relatively large deflections owing to a significantly smaller diameter to overhang length ratio. This results in a drastic reduction in tool shank section modulus, which lowers its strength and ability to withstand periodically varying cutting forces, leading to tool bending [85]. The increased flexibility and lower stiffness of smaller diameter tools result in large values of cutter edge deflections, which lead to two serious problems: form and feature geometric errors on the machined component and distortion of cutting forces. This effect is again strengthened even further as the cutting tool diameter reduces from 1 000 µm to 100 µm, where even a small deflection of 5 µm will lead to an error comparable to the cutting edge radius of the tool. As the deflected tool rotates, undesirable cycles of shearing to ploughing modes of material removal mechanism will occur, leading to spikes in high and low cutting forces on each tooth. This will in turn cause larger deflections, while the cycle itself will continue until either failure of the tool occurs or the tool skips. Moges et al. [85] presented a methodology for determining such cutting force-induced tool deflections and developed a cutting force model considering tool deflection on the resulting cutting forces. Similarly, Mamedov et al. [87] developed a novel mathematical model for estimating cutting force and tool deflection by calculating the UCT, which considered both ploughing and shearing modes of material removal. On the other hand, Lu et al. [88] proposed a revised 3D analytical model of micro-milling forces, which considered the effects of cutting temperature and ploughing force caused by the arc of the cutting edge during shearing-dominated cutting. Therefore, considering the seriousness of the tool deflection issue, it is of great importance to study its effect on the mechanics of the chip formation process, while examining cutting forces, surface errors, and cutting temperature, so that reliable and accurate predictions can be made to limit and prevent excessive deflections during machining.

Cutting forces directly affect tool deflection in the micro-milling process because of the relatively low stiffness of the tool, particularly at the tool tip, and results in imperfections on the machined surface as described above. As bases for determining tool deflection, accurate analytical cutting force models that consider the tool geometry and material, the specific cutting mechanism involved, as well as the vibration dynamics are key areas for research. Mamedov et al. [87] fully understood the importance of cutting force on tool deflection and became significant contributors to micro-milling tool deflection analysis early on. Using their mathematical model, the distribution of forces acting on the tool can be predicted and deflection of a micro-milling end mill tool can be estimated with good accuracy. High cutting forces lead to higher tool deflection. Mamedov et al. [89] presented an updated analytical cutting force model, which considered both the shearing and ploughing phenomena, based on the material elastic recovery properties. The tool deflections corresponding to the cutting force were calculated by considering the microtool stiffness. This model accurately predicts instantaneous tool deflections through analysis of the cutting force, which was presented as a function of cutting force coefficients, microchip thickness model, and tool geometry. Oliaei and Karpat [90] investigated the influence of increased cutting force due to tool wear on tool deflections and tool breakage. Their model for predicting tool deflection and tool breakage allows for the development of tool condition monitoring systems based on the physics of the micro-milling process. In their model, Rodríguez and Labarga [91] for the first time considered variable deflection rather than just static deflection along the cutting edge. Their model also has promising benefits in monitoring systems and adaptive control systems for the prevention of tool failure during micro-milling operations; however, it does not take tool wear into consideration. Moges et al. [85] also presented a method for determining cutting force-induced tool deflections and developed a flexible force model considering the effect of tool deflections on the resultant cutting force based on previous rigid models. The team presented a methodology for predicting variation in machine surface error due to tool deflections. Their proposed model accurately predicted cutting forces in the presence of tool deflections. In addition, it was found that deflection of the tool caused considerable deviations of the tool center location, resulting in change of tooth trajectories and uncut chip geometry. Their model provided great benefits in selecting optimum cutting parameters to control tool deflections, resulting in tighter tolerances and improved productivity. However, to further improve the prediction accuracy, their model must consider the dynamic vibration of the tool tip. Lu et al. [92] understood the importance of examining the cutting force and how it might be used to limit tool deflection. They proposed an indirect method of determining the average micro-milling cutting force, which was both low cost and high precision, by examining the power of the main transmission system of a micro-milling machine. Lu et al. [93] then developed a new method for predicting micro-milling tool breakage based on theoretical models by examining the tool bending stress. Finally, Zhang et al. [94] formulated a mechanistic model of cutting forces and instantaneous tool deflection in the micro end milling process, which took into account the minimum UCT effect and tooth trajectory. Their model also considered tool runout, consisting of both axial and tilt offsets, including entry and exit angles of the tool. Their developed model can be used to further optimize the accuracy of the micro-milling process because of the inclusion of a more complete tool deflection model. Clearly, deflection of a cutting tool is dependent on many factors and must be modeled as a dynamic phenomenon, rather than a static one. More accurate tool deflection prediction models will provide methods for reducing cutting forces, thereby reducing tool wear and breakage, increasing surface and feature quality as well as machining efficiency. However, tool deflection is an implicit issue of machining at the microdomain, which also has a multifactor influence on the process stability as a whole, similar to tool runout and self-excited machining chatter. Therefore, a thorough review of relevant research is presented below on tool runout and chatter in micro-milling.

### Tool runout

**Fig. 8:**
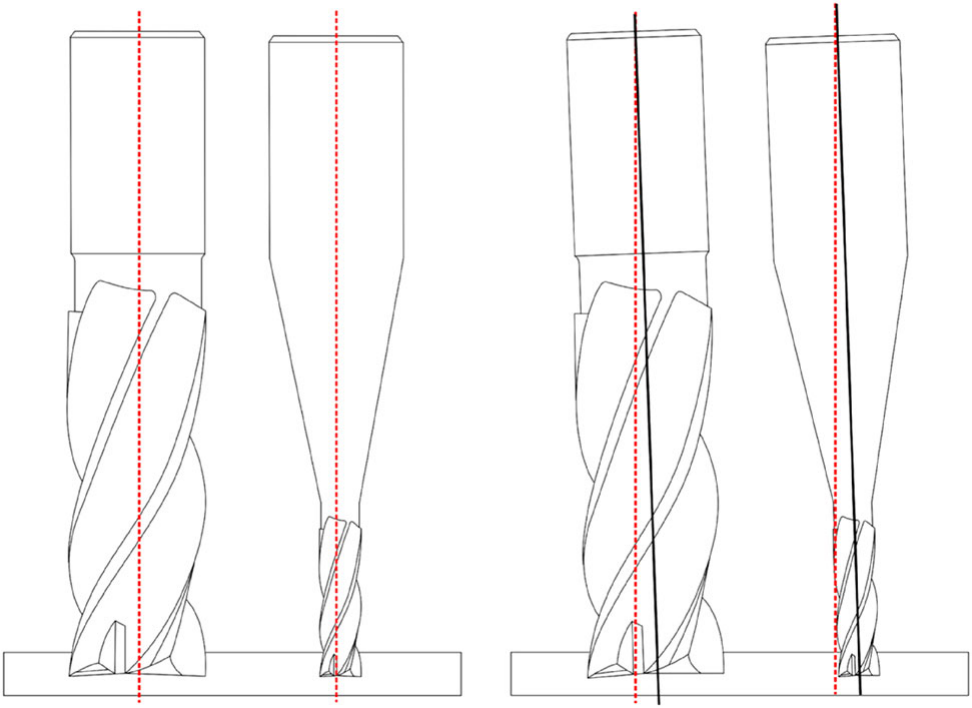
Effect of runout is intensified for smaller tools (Reprinted from “Protocol for tool wear measurement in micro-milling” by Alhadeff et al. [63], with permission from Elsevier)

Tool runout is a critical issue that affects the micro-milling process significantly. It is in part responsible for influencing the cutting force [96], tool condition, tool life [97], and surface integrity of the machined component [98]. Tool runout can be described as a phenomenon caused by the sum of the geometrical displacement errors of the spindle, toolholder, and tool axis from the ideal or theoretical axis of rotation. The sum of these errors produces a deviation between the theoretical and actual cutting edge trajectories [62]. Tool runout may take the form of axial and/or radial runout. Radial runout is caused by the tool rotating off center, instead of being centrally aligned, and it will rotate about a secondary axis. Cutting tools will be generally more tolerant to this type of runout during face milling operations. However, during side milling, radial runout will have significant effects on the cutting force, and therefore tool wear, due to uneven loading on the flutes, which will lead to surface errors. In contrast, axial runout is the result of rotating components not being parallel with the center axis of rotation, such as the tool axis and spindle axis not running concentrically. Therefore, axial displacement of the tool causes its tip to rotate off center relative to the spindle axis. Cutting tools will generally be less tolerant to axial runout, especially for micro-milling operations in both side and face milling operations, but axial runout has a considerable influence on the surface topography generation in face milling [99]. The total tool runout is therefore the sum of both axial and radial runouts, with the effect becoming even more significant in the micro-milling domain, as demonstrated by Fig. 8. Because micro-milling requires very high spindle speeds due to the relatively small cutting edge diameters, the dynamic characteristics of the spindle-tool system dominate the machining process quality. Therefore, tighter stiffness loop machines with higher precision spindles and tools are essential. However, even with the correct equipment, the tool-spindle interface can cause undesirable radial runout, while even small deviations in the spindle or cutting tool edges may result in significant runout due to poor stiffness and strength of microtools [84].

The significance of tool runout is that it has a major influence on the cutting force. This is due to the displacement being in the same order of magnitude as the feed per tooth, which therefore has a large influence on the surface roughness generated, as determined recently by Chen et al. [99]. They also found that axial runout in particular limited the achievable surface roughness. Similarly, uneven engagement of teeth caused by runout leads to uneven rates of wear on each tooth, resulting in cutting force features that can be largely different for both teeth [63]. This further leads to increasing cutting forces and all problems generated by process instabilities. Attanasio [62] developed an easy and reliable method for determining tool runout in micro-milling by implementing a geometric model that deduced and estimated tool runout from the tool diameter, channel width, and cutting edge’s phase. Their procedure can be integrated into an adaptive model for controlling cutting force, which has practicality for improving production quality and process stability while reducing tool wear and machining costs. Li et al. [15] established a cutting force model that further strengthened the understanding of the micro-milling process, through a deeper investigation of tool eccentricity. This multifactor model considers the influence of runout on the UCT, the equivalent rake angle and cutting force, and how the combined effects of each factor influence the surface quality.

With regard to measurement of tool runout, Jing et al. [100] presented a method using modeling and simulation of the cutting force in micro-milling. The proposed approach uses a charge-coupled device to determine differences in displacement of tool flutes and tool shank. An accurate tool runout value can then be calculated using their model. This is a simple, easy, and precise method for measuring runout in micro-milling and can be easily adapted to on-machine measurements during operation. Another simple method for measurement of tool runout is by displacement measurement using capacitive sensors close to the tool shank, according to Chen et al. [101]. They also determined that tool runout resulted in a considerable increase in surface roughness, particularly when the feed per tooth was less than the runout. Finally, their proposed surface generation model considering the minimum UCT, which takes into account tool runout, provides a more accurate surface topography simulation and roughness prediction in micro-milling. Zhang et al. [102] also developed a simple and effective tool runout identification method, designed to quickly identify the tool runout parameters through tool displacement measurement using a laser displacement sensor, so that the accuracy of tool runout measurements could be improved.

Guo et al. [103] established the importance of a more systematic approach to investigating tool runout in relation to tool geometry and surface generation. Toolpath optimization during 5-axis machining was examined in detail in relation to minimizing geometric errors formed from tool runout. In their model, the tool runout is defined by four parameters, namely, inclination angle, location angle, offset value, and length of the cutter axis. Although their work only considered conventional machining, much of what was learned could also be applied to micro-milling, with the effect becoming even more significant at the microdomain. Guo et al. [104] then presented an instantaneous UCT model regarding tool runout and tool geometry in micro-milling. Using their early work as a foundation, they determined that five parameters were necessary to characterize tool runout in micro-milling, namely, runout offset length, inclination angle, cutter axis length, location angle, and initial rotation angle. The team analyzed and discussed the influencing principles of each runout parameter on the instantaneous UCT values. Some important viewpoints that provided reasonable explanations for each runout parameter were introduced. However, no method for detecting each of the runout parameters was offered. Their work would be a good research to begin more thorough investigations into fully characterizing the tool runout parameters and how this phenomenon could be minimized or eliminated to reduce cutting forces. As mentioned, tool runout causes unbalanced chip thickness removal between flute teeth, which leads to uneven cutting force loading on each cutting edge. This causes not only unwanted vibrations that affect process stability, but also high and uneven tool wear. Throughout this section, it is explained why it is important to limit tool runout as much as possible. However, it may not always be feasible to completely eliminate runout owing to a number of reasons including tool, toolholder, and spindle setup, and tool manufacturing tolerances. Therefore, it is essential to limit tool runout to an acceptable level, i.e., at least below 2 µm. This can be considered prior to machining to realize a precise and accurate process, avoid accelerated tool wear or tool breakage, and improve surface finish.

### Chatter

Chatter also greatly influences the process stability, resulting in increased tool wear, poor surface finish, and limiting precision and efficiency. Chatter is a form of self-excited, unstable vibration during specific cutting edge machining. It is generally accepted that there are four types of chatter during the cutting process, namely frictional chatter, regenerative chatter, mode coupling chatter, and thermomechanical chatter [105]. Frictional and regenerative chatters are generally the most common types and notably most important in micro-milling. Frictional chatter is mainly attributed to nonlinear dry friction force, i.e., rubbing on the clearance face, which leads to excitation vibration of the cutting force, limiting the thrust force [106]. However, regenerative chatter is the most significant issue in cutting processes in general because of the high spindle speeds involved [105]. It occurs owing to varying cutting forces acting on each tooth of the tool, which create a relative displacement between the tool and the workpiece at the cutting point [107]. Depending on the characteristics of the system and the phase between the varying cutting forces, the dynamics of the cutting system can be unstable. This in turn leads to large chip sizes and higher cutting forces and vibrations. This process will continue if the system remains in an unstable condition, until the vibration amplitude increases to the point that the tool jumps or skips, damaging either the tool, workpiece, or spindle [108]. Therefore, to prevent chatter and unstable machining conditions, accurate models of the dynamics of the micro-milling system are necessary to predict the relationships between the workpiece material, structural dynamics of the machine tool including toolholders, tool geometry, and cutting conditions. By analyzing these models, a stability lobe diagram (SLD) can be created, which will offer insights into ideal machining parameters that can be chosen to prevent process instabilities such as chatter, greatly improving the machining efficiency [109].

**Fig. 9:**
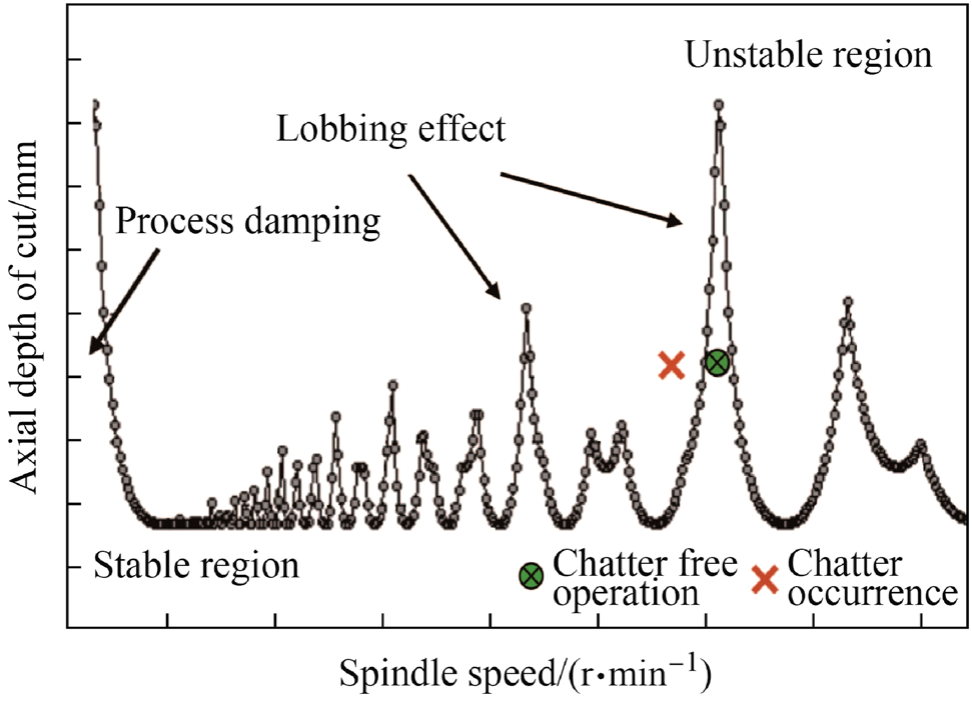
Stability lobe diagram plotting axial depth of cut against spindle speed to identify areas of chatter-free operation. Reprinted from “Chatter in machining processes: A review” by Quintana and Ciurana [105], with permission from Elsevier

**Fig. 10:**
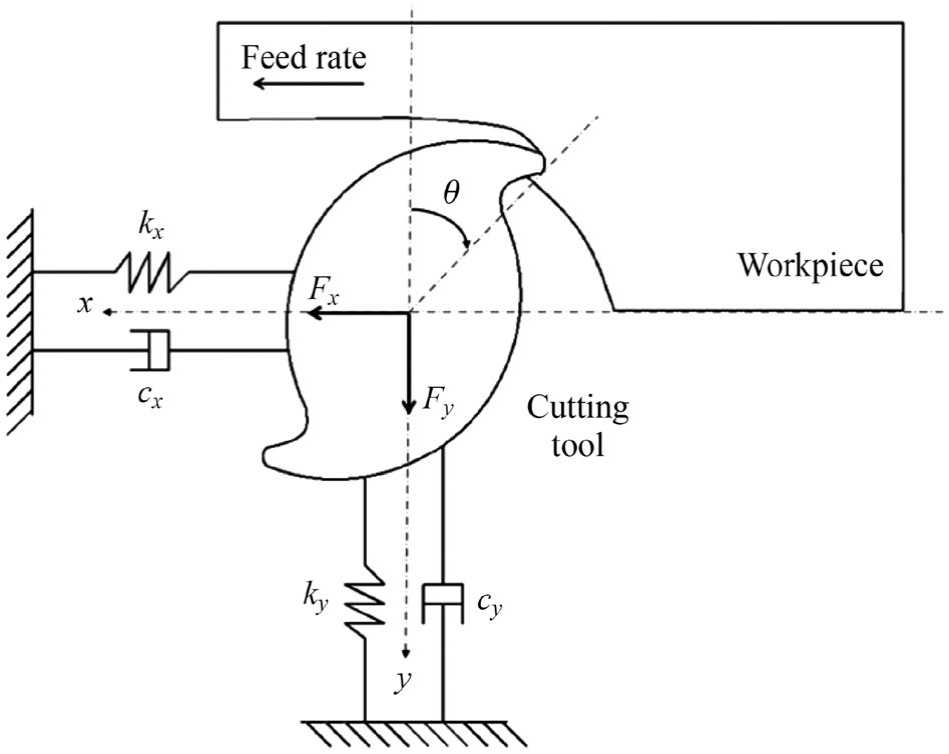
Dynamic model of micro-milling system showing stiffness loop and how chatter can be modelled (Adapted and reprinted from “Chatter modeling in micro-milling by considering process” by Afazov et al. [98], with permission from Elsevier)

The distinction between a stable and unstable cut can be visualized with the SLD, which plots the axial depth of cut as a function of the spindle speed, as depicted in Fig. 9. This diagram is an essential tool to find the range of machining parameters that results in a maximum stable (i.e., chatter free) material removal rate [110]. The idea is to seek regions within the lobes for optimal machining parameters, depending on such criteria as time, cost, and accuracy [105]. However, SLD is based on individual machine setups as indicated in Fig. 10, which considers the machine tool stiffness loop, tool geometry, etc. Therefore, predicting the stability lobe boundaries can be a very difficult task that will rely on fundamental understanding of the dynamics of the entire micro-milling process. To do so, a combination of theoretical models of the machine tool and toolholder, as well as deflection testing of the tool will be necessary. To begin constructing an SLD, an analytical model of the frequency response function (FRF) of the cutting tool, toolholder, spindle, and machine tool is required. Next, experimental testing or an accurate theoretical model is required to determine the dynamics of the tool tip. Thus, SLDs can be created for a system setup using the specified cutter, workpiece material, etc. Finally, the operator can select combinations of axial depth of cut and spindle speed, which ensure chatter-free operation.

In contrast to that of conventional milling tools, performing an impact hammer test at the tool tip of micro-milling tools is not feasible because of their inherent tool fragility. Therefore, new methods for analyzing micro-milling tool dynamics are necessary to build the SLD in micro-milling. Lu et al. [111] developed a vibration displacement measurement system that utilizes a laser displacement sensor to collect vibration signals during micro-milling. The frequency of the micro-milling cutting force was obtained using the varying cutting parameters method, whereas the relationship between the cutting force amplitude, frequency, and vibration displacement was ascertained by using a neural network method to realize vibration displacement prediction under given cutting parameters. Before this important work, the methods used for conducting stability analysis mainly included zero-order solving, semi-discrete, and time domain methods [112]. The zero-order solving method was applied by Mascardelli et al. [113], Tajalli et al. [114], and Jin and Altintas [115]. However, only stability prediction results considering the shear effect were obtained for the SLDs drawn. Tyler et al. [116] presented a method for producing SLDs that included process damping ranges (low cutting speed) and high cutting speeds. This method defined the stability boundaries by radial rather than axial depth of cut, because of the approach taken by computer-aided modeling toolpath programs. This work is particularly significant in defining machining parameters for difficult-to-machine materials. For these materials, high tool wear prohibits high spindle speeds, which therefore leads to smaller stable zones in the SLDs. Park and Rahnama [117] obtained micro-milling tool tip dynamics indirectly through mathematical coupling of the substructures using the receptance coupling method. Song et al. [118] and Tajalli et al. [119] drew an SLD using a semi-discretized numerical approach to predict chatter stability based on cutting force. However, the authors noted that further study was necessary to investigate how the burr formation mechanism would affect the process stability, which led to chatter. Lu et al. [120] provided a clear basis for the dynamic study of the tool-toolholder-spindle system based on receptance coupling substructure analysis and by considering rotational degree-of-freedom (DOF) and tool point FRF of micro-milling. The FRF at the micro-milling tool point can therefore describe the dynamic behavior of the entire micro-milling machine system. Lu et al. [121] further developed this work by considering the centrifugal force and gyroscopic effect caused by the high-speed rotation of the micro-milling spindle to better simulate the real scenario and increase the accuracy of modal parameters.

To obtain more accurate SLDs, higher accuracy models of chatter and process stability are necessary. Typically, models designed based on solving the equations of motion in either the frequency or time domain, where both cutting force and modal parameters are implemented, will lead to results that are more robust. This is because “cutting instability consists of deterioration in both time and frequency domains due to the highly nonlinear nature of the micro-milling process” [122]. When the cutting forces exhibit a linear behavior in cutting processes, the frequency domain solution should be used, i.e., when the process is more or less stable. However, at small UCTs and feed rates, the micro-milling process experiences nonlinear behavior owing to the size effect, chip formation, etc. Since the cutting forces can become nonlinear, the equations of motion must therefore be solved in the time domain using numerical methods for integrating the ordinary differential equations of motion [98]. The dynamic system can then be reduced to a 2-DOF system through the assumption that the helix angle of the microtool is negligible, simplifying the equations of motion (see Fig. 10). Regarding the time domain method, Lu et al. [112] proposed a micro-milling force prediction model based on chatter stability analyzed in the time domain. However, because the time response is bounded, the process can become significantly unstable and chaotic in the frequency domain, which can lead to geometric errors and tool damages due to chatter. Liu et al. [122] developed a novel simultaneous time-frequency control theory to regulate and counteract the various nonlinear dynamic instabilities including chatter and tool resonance. This model controls the dynamic response of the system under various axial depths of cut and spindle speeds to prevent an unstable state of motion. The simultaneous time-frequency control model was found to demonstrate the capability of reducing chatter and process instabilities during micro-milling operations, leading to improved tool performance and machined surface quality.

Chatter worsens the machining precision and efficiency, as well as tool and surface integrity [123]. By understanding the dynamics of the system through analysis of the cutting forces and developing accurate prediction models, the effect of chatter on the micro-milling process can be minimized, if not entirely eliminated. Using SLDs, suitable machining parameters can be chosen to avoid chatter and unstable machining conditions. To obtain such diagrams, it is suggested that both time and frequency domains should be considered simultaneously to control the process.

## Process inputs

As with any manufacturing process, the input variables, such as workpiece material, tool parameters, toolpath, and cutting fluid, are all widely known to affect the process outputs of cutting force, surface quality, tool wear, etc. The influence and multifactor effect of the process inputs have even more significance at the microscale, particularly when the microstructure of the tool and workpiece must be considered and they are of major concern to the micro-milling process performance. Therefore, it is essential to fully understand the process physics and characterize the effect of each variable in a systematic way, so that the input parameters that have considerable influences on the output quality can be easily recognized and accounted for. In theory, the optimization of the process for all machining variables appears to be very complex. However, considerable achievements focusing on predictive models, numerical simulations, statistical analysis, and experimental investigations have been made recently, such that the multitude of factors influencing the process outputs can now be examined accurately. Therefore, a full review of existing investigations on input variables and their effect on the micro-milling process outputs is presented.

### Process outputs

Firstly, a brief introduction and investigation of major process outputs are necessary to understand how they are influenced by process inputs by examining the predictive models, as well as theoretical and experimental works presented recently, so that the optimal micro-milling process can be determined for a range of difficult-to-machine materials. Beginning with the cutting force, it has been shown that typically within the range of selected cutting parameters, the spindle speed has a relatively weak influence on the cutting force, while it tends to increase initially when the feed per tooth is close to the radius of the cutting edge, leading to a dominant ploughing mode of material removal. It then tends to decrease almost linearly with a further increase in feed per tooth, while the shearing mode of material removal dominates [74, 88, 124]. The cutting force also clearly increases with an increase in depth and width of cut, as does the cutting temperature. In this regard, the cutting temperature also tends to increase at first but then decreases with the increase in feed per tooth, where the turning point is at the UCT. In contrast, the cutting temperature increases considerably with the increase in spindle speed [88, 125]. Another important parameter to evaluate the effectiveness of the micro-milling process is surface quality, often examined as surface roughness, burr formation, and remaining artefacts of the micro-milling tool on the machined surface. Lu et al. [126] established a comprehensive floor surface model to predict both the surface roughness and such artefacts or grooves under different cutting parameters and tool parameters. It was found that surface roughness decreased first and then increased with the increase in spindle speed, but it increased with the increase in feed per tooth and depth of cut. However, as noted by the authors, to obtain the individual and combined interaction effects of each cutting parameter and how they influence the process outputs, more experimental data are required. Lu et al. [127] further developed this study through an analysis on the effects of spindle speed, radius of a ball end mill, axial cutting depth, and feed per tooth on the curved surface roughness. Moreover, they also built a surface roughness prediction model to provide an accurate reference for the selection of cutting parameters in the micro-milling of Inconel 718.

All of this important research work concerning micro-milling has been carried out with the goal of advancing the micro-milling process in terms of efficiency and productivity, so that it may develop new applications across new industries. Therefore, a method for quantitatively analyzing the efficiency during experimental work is essential to determine the progression of the micro-milling process. The material removal rate, which is a measure of the amount of material removed per unit time when performing machining operations, is often used to do so. Lu et al. [128, 129] established an optimization approach based on genetic algorithm to achieve the maximum material removal rate under the constraints of surface roughness and cutter breakage. Peng et al. [125]. determined that when the rate of material removal was the same, a higher spindle speed was better for reducing surface deformation. Similarly, when the spindle speed is the same, a higher material removal rate is better for reducing deformation. To improve the machining efficiency, selecting a high spindle speed and feed rate has a great significance in promoting the workpiece quality in micro-milling. An undesirable process output that can occur in metals with a crystal structure is work hardening, also known as strain hardening. The strengthening of the material is due to dislocation movements and dislocation generation within the crystal structure of the material when it is strained beyond its yield point. An increasing stress is then necessary to produce additional plastic deformation, leading to significant tool wear, higher cutting forces, higher cutting temperature, and overall lower machining efficiency during micro-milling. Lu et al. [130, 131] used 3D FE analysis for simulating the process of micro-milling to predict the surface hardness of both Inconel 718 and a nickel-based superalloy. With regard to other process outputs, the team then studied the influence of cutting parameters, including the spindle speed, feed rate per tooth, and axial cutting depth on surface Vickers hardness, as well as the relationship between strain and hardness [132, 133]. According to their analysis, the spindle speed has the greatest influence on Vickers hardness, whereas the axial cutting depth has an intermediate influence, while the feed per tooth has the least influence. Their work can help guide the selection of cutting parameters to reduce surface work hardening, and thereby improve the quality of the final product. Clearly, selection of appropriate cutting parameters prior to micro-milling operations is essential for improving machining efficiency and quality, prolonging the tool life and maintaining good surface quality. However, the micro-milling process can only be optimized to a certain degree through selection of machining parameters. Therefore, a detailed investigation and discussion of the other major process inputs, namely the workpiece microstructure, the microtools themselves, the toolpath, and cutting fluid, are necessary to develop better understanding of the process as a whole.

### Workpiece microstructure

The influences of the microstructure of multiphase materials and the process outputs in micro-milling require very detailed investigations to accurately develop robust analytical models, because the workpiece can no longer be described as homogeneous at the microscale. Better models and understanding of the material microstructure and its machinability will help develop more accurate predictive models to avoid tool wear, improve surface quality, etc., during the process design and machining phases [39]. Clearly, the anisotropic behavior of multiphase material microstructures is an important factor that must be considered throughout the machining process when the size effect and chip formation mechanisms are influential at the microscale.

Vogler et al. [134] presented a very early significant mechanistic model for the micro-milling process that explicitly accounted for different phases of heterogeneous materials. This model explicitly considers the multiple phases and the effect of determining the magnitude and variation in cutting force. The team showed that the microstructural effects could account for more than 35% of the energy in the cutting force signal. Attanasio et al. [135] also investigated the influence of material microstructures on the cutting force, with a detailed examination of four different microstructures, namely bimodal, fully equiaxed, fully lamellar, and mill annealed, of Ti-6Al-4V alloy. The team observed lower cutting forces, lower BUE, and reduced tool wear for the fully lamellar microstructures. Understanding the variation in cutting force is essential in developing a more complete model of the excitation and process stability between the tool and the workpiece. This will lead to further development of the micro-milling process as a whole and offer insights into better microstructure design when micro-milling. With regard to surface roughness, Elkaseer et al. [136] presented a model to simulate the surface generation process in micro-milling of multiphase materials. They confirmed that their developed model could be used to predict the surface quality after machining under various machining parameters and could further be used to optimize the process for multiphase materials. An important feature of the model is that it considers micro-burrs at the phase boundaries.

**Fig. 11:**
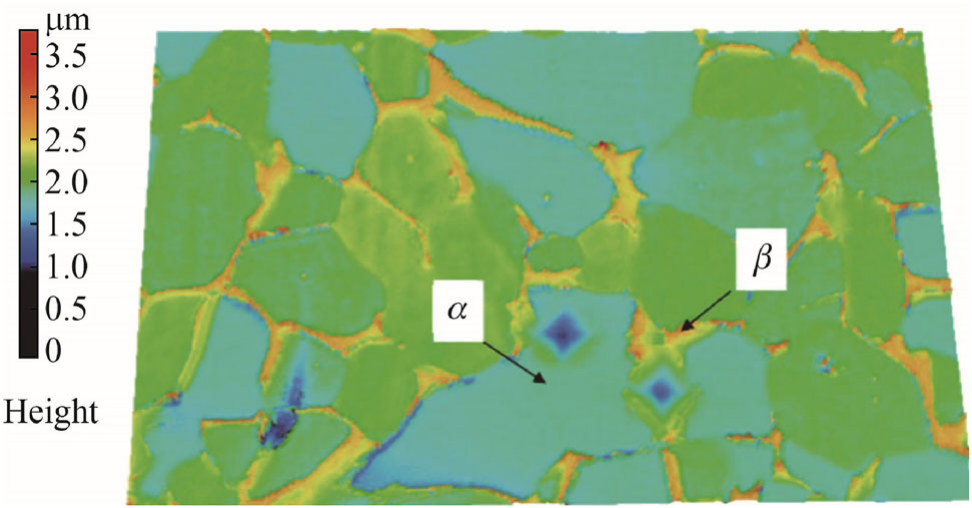
Indentation marks on α and β phases showing relevant depths of indentation, which are used to identify areas of different microhardness due to phase change (Adapted and reprinted from “Microstructure effects on process outputs in microscale milling of heat treated Ti-6Al-4V alloys” by Ahmadi et al. [39], with permission from Elsevier)

Concerning workpiece microstructure characteristics, Ahmadi et al. [39] investigated the influence of grain size, grain boundary, and phase fractions in the micro-milling of Ti-6Al-4V on the process outputs. A smaller grain size (both *α* and *β*) and lower *β* phase fraction led to a higher cutting force in micro-milling. Although, the hardness of the sample containing enlarged equiaxed grains was found to be higher owing to the greater *β* phase fraction as displayed in indentation tests (see Fig. 11), it experienced a lower cutting force as a result of its lower ductility. Moreover, the team found that the microstructure could greatly affect the BUE formation in terms of size and shape; therefore, lower grain sizes can result in more BUEs. Aksin and Karpat [137] also investigated the influence of microstructure on the process outputs as a function of grain size and grain morphology on commercially pure titanium using their developed mechanistic model. The microstructure was modified using heat treatment methods, so that a gradual transition from acicular to equiaxed grain morphology was obtained. They also established that as the microstructure becomes more equiaxed, the hardness increased. However, unlike those of Ahmadi et al., their results showed increased cutting forces in this case. Elkaseer et al. [138] examined the effects of material homogeneity of copper (Cu99.9E) on the minimum UCT and showed that by refining the material microstructure, the minimum UCT could be reduced. It was also confirmed that material homogeneity improvements led to a reduction in surface roughness and surface defects in micro-milling.

It is evident that the resulting surface integrity after micro-milling is highly dependent on the material microstructure, especially for multiphase materials. Therefore, deeper consideration of the cutting conditions and material microstructure must be given prior to micro-milling operations, and the analytical models introduced above can help significantly. Good understanding of these relationships will lead to future development of more accurate microstructure-based predictive models of the micro-milling process based on computational techniques.

### Microtools

The continuing trend toward smaller feature sizes in micro-milling with higher precision and accuracy has led to demands for higher quality microtools, as the cutting tool edge radius defines the minimum UCT [139]. It was shown by Kirsch et al. [140] that the material specifications of tool blanks highly influenced the quality and application of ultra-small microtools in the range of 4–50 µm. Generally, microtools are manufactured via grinding operations following the famous procedure by Aurich et al. [141] for the design and machining of single-edge micro end mill tools with diameters between 10 µm and 50 µm and a variable helix angle. Cemented carbides, such as tungsten carbide, are predominantly used as micro-milling tool materials owing to their high stiffness, hardness, and resistance to wear. It was shown how sharper and more homogeneous cutting edges without breakouts might be achieved with smaller grain sizes of cemented carbide, while the application of these tools generated smaller cutting forces and resulted in a considerably longer tool life. The quality of the manufactured tool may depend on the material, overall geometry, cutting edge radius, surface conditions, and coating, while the tool design influences the dimensional accuracy, surface quality, burr formation, and tool life [142]. Therefore, it is extremely important to thoroughly investigate all possible factors and influences that the micro-milling tool may have on the machining process.

In relation to microtool geometry, Kirsch et al. [140] discovered that although their 50 μm diameter cutting tool provided defined sharp cutting edges and faces that appeared smooth, the cutting edges of the 10 μm diameter tool appeared blunt and grinding grooves could clearly be observed. This shows that the tool diameter cannot be reduced by only scaling the tool geometry, which supports the conclusion that the microgeometry and microstructure of the tool must be adapted to accommodate even smaller diameter cutting edges. Finally, the team determined that the machining parameters must also be optimized to ensure a high-quality cutting edge surface and overcome the size effect. Similarly, Cheng et al. [142] presented a thorough study on micro-milling tools, noting how commercially available tooling was generally downscaled from macro-milling tools that were not accurately fabricated nor entirely suitable. Therefore, the team proposed a design criterion for custom micro-milling tools and developed a new micro-hexagonal end mill, fabricated using wire electrical discharge machining (EDM) based on their considerations. Their developed tool achieved submicron surface roughness values for the side and bottom surfaces. Most importantly, Fang et al. [143] determined that the tool tip rigidity of a semi-circle-based (D-type) end mill was much higher than that of a two-flute (commercial type) end mill. This shows that tool geometry plays a major role in tool stiffness in micro-milling, which is important in reducing tool deflection, cutting force, and therefore tool wear.

Adhesion of material on the cutting edge of microtools can quickly lead to surface quality deterioration, as reported by Katahira et al. [144], who performed ultra-precision machining of a single crystalline sapphire using a polycrystalline diamond (PCD) micro-milling tool. To restore the milling capabilities of the PCD tool, the team implemented an electrochemical-assisted surface reconditioning process to remove the surface contaminant and restore the machining performance of the PCD micro-milling tool. Adhesion of material on the cutting edge of microtools can be prevented by coatings. It was shown by Swain et al. [145] through a direct comparison between TiAlN-coated and uncoated tungsten carbide micro-milling tools that TiAlN-coated tools exhibited superior performance in terms of tool life and micro-burr formation. On the other hand, Thepsonthi and Özel [146] attempted to improve the performance of carbide micro-milling tools by applying a cubic boron nitride (cBN) coating to the end mill tools. Their study clearly showed that the cBN-coated carbide tool greatly outperformed the uncoated carbide tool in terms of tool wear and cutting temperature.

**Fig. 12:**
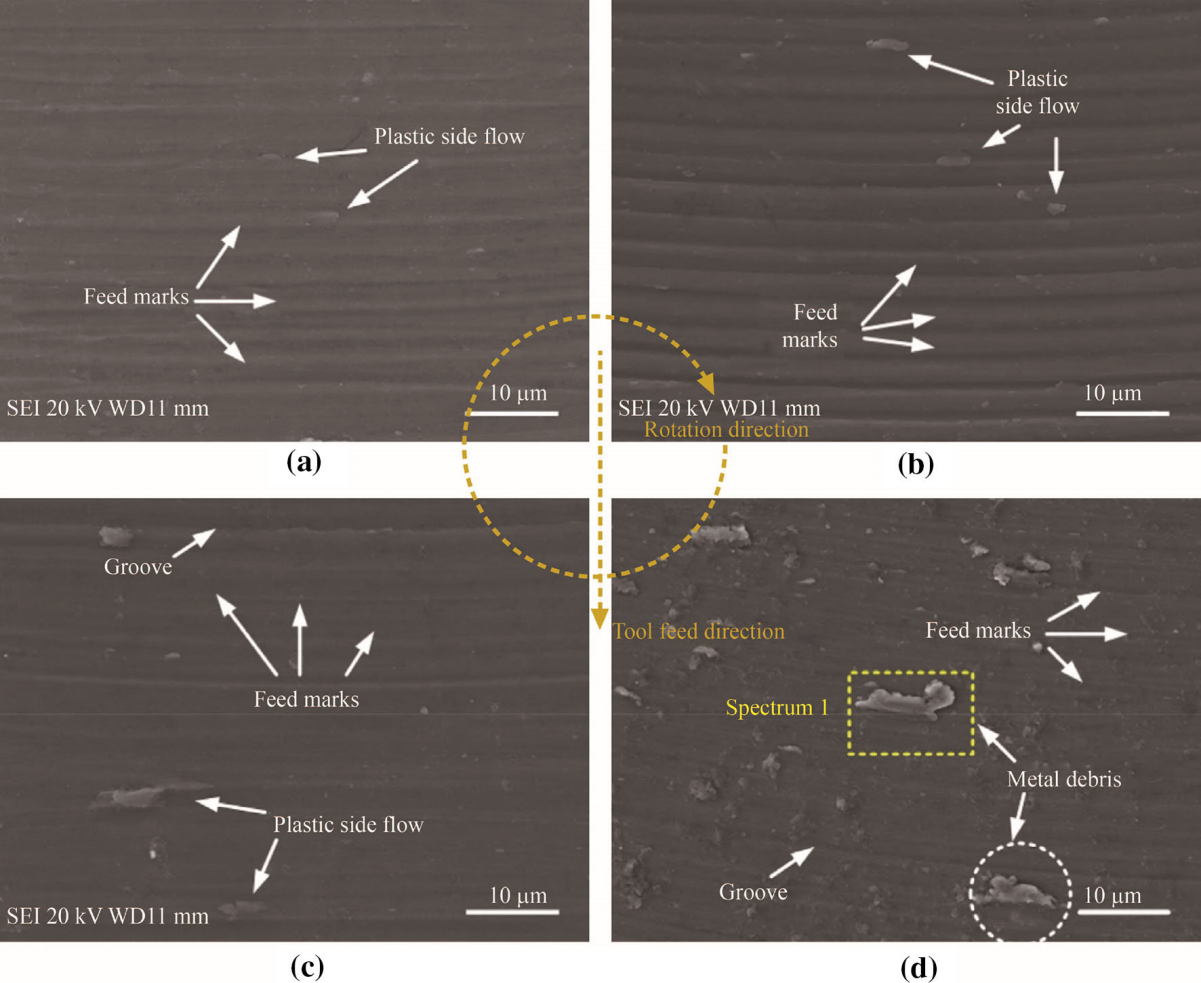
Surface topography of the micro-slot middle region at the cutting length **a** 1 mm, **b** 166 mm, **c** 249 mm, and **d** 332 mm (Adapted and reprinted from “Effect of the progressive tool wear on surface topography and chip formation in micro-milling of Ti-6Al-4V using Ti(C7N3)-based cermet Micro-mill” by Wang et al. [150], with permission from Elsevier)

As for recent advances in tool materials, Suzuki et al. [147] developed and manufactured micro-milling tools from binderless ultra-hard nano-PCD (NPCD) to machine silicon carbide (SiC) molds using laser fabrication techniques. The NPCD consists of very fine grains having a length of several tens of nanometers and is harder and more thermally stable than conventional PCD. It was demonstrated that the tool wear was exceedingly small compared to that of PCD tools, while a microtextured surface with very fine textures was created on the SiC mold in the ductile mode of material removal. Another material commonly used for micro-milling tools is chemical vapor deposition (CVD) diamond owing to its exceptional hardness and wear resistance. However, the fabrication of such tools by conventional grinding processes is inefficient in terms of productivity and edge quality. Yang et al. [148] developed a novel hybrid machining process that combined laser-induced diamond graphitization with precision grinding to attain high-quality CVD diamond micro-milling tools. Zou et al. [149] presented a micro-milling investigation of Ti(C_7_N_3_)-based cermet tools developed in-house, taking into account the wear forms and wear mechanisms [150]. Their results showed that adhesive wear and microchipping were the main wear mechanisms of the major and minor cutting edges, respectively. It was also demonstrated that metal debris and plastic side flow became more severe as the tool wear progressed, as shown in Fig. 12. On the other hand, surface quality at the up-milling side was better than that at the down-milling side.

Currently, the smallest commercially available micro-milling tools have diameters of 50 µm, with minimum achievable machined features depending on workpiece material, machine tool accuracy, and feature geometry [151]. Therefore, further research and more in-depth investigations will be necessary to explore the geometry of more efficient tools, examine the limits of tool and feature aspect ratio, and move toward submicron tool diameters in the distant future. In addition, the influence of ultra-thin coatings and tool reconditioning processes on micro-milling tools will be interesting areas for further research in the future.

### Toolpath

Thepsonthi and Özel [152] proposed an integrated method for selecting both toolpath and optimum process parameters to meet certain machining requirements and constraints. The method outlined considers a mathematical model for determining the optimum toolpath strategy by using data from their experiments and FE simulations. Optimal toolpath and process parameters can be used to establish more accurate predictive models by considering and maintaining an acceptable tool-workpiece engagement load. However, it was verified that the resultant optimal toolpath strategy could only determine a certain level of process performance, while a more in-depth study would be needed to ensure burr free micro-milling. Finally, the team demonstrated that the toolpath strategy strongly affected tool wear and burr formation, while FE simulations provided an effective platform for toolpath selection.

An analysis of micro-milling vibration minimization and surface quality was presented by Wojciechowski and Mrozek [153], who used ball nose end mill tools at various tool axis inclination angles along the toolpath. They showed that the tools axis of inclination in the direction perpendicular to the feed motion significantly affected both the dynamics of the process and the surface quality. Decreasing the inclination angle caused nonlinear growth of vibration amplitude and surface roughness. The findings were attributed to the ploughing-dominant regime resulting in growth of the cutting edge forces at low angles of inclination.

For thin-walled structures less than 100 µm, it was found by Annoni et al. [154] that the down-milling strategy was more influential with regard to geometrical errors, such as flatness deviation and average thickness error, compared to the up-milling strategy. Their results also showed that the toolpath factor did not influence the geometrical response. However, they recommended the application of step support, i.e., removing material from either side of the wall in an offset technique and using the up-milling strategy. Zariatin et al. [155] determined that there was no specific correlation found among spindle speed, feed rate, and machining strategy with the thin-wall accuracy [155].

Regarding path strategies, Koklu and Basmaci [156] presented a study on the influence of cutting path on the cutting force and surface quality during micro-milling pocket operations through analysis of the hatch zigzag and contour climb toolpath strategies under different cooling conditions. It was revealed that better results of up to 40% reduction in cutting forces and better surface quality were obtained with the use of the contour climb, also known as down milling, compared to those of the hatch zigzag strategy for AA 5083 H116 aluminum alloy. These results contradicted those of Annoni et al. [154] who recommended conventional, also known as up milling, on 0.4% carbon steel (C40).

In milling of freeform surfaces using ball nose end mill tools, it may not always be possible to maintain the inclination angle so that the tool center is taking part in the cutting process. Therefore, depending on the toolpath, this may lead to two material removal regimes, i.e., where the cutting edge removes material by the shear mechanism and where the tool center extrudes material through the ploughing mechanism, as investigated by de Souza et al. [157]. Again, this effect will be even more substantial at the micro-milling scale, especially leading toward freeform micro-milling. Similarly, modeling the cutter envelope surface is another important aspect as it can be used to predict geometric errors and optimize toolpaths in conventional machining, according to Guo et al. [103]. Therefore, compensating tool runout errors during micro-milling in toolpath planning may be helpful for maintaining process stability in the future for 5-axis micro-milling. It is very surprising that toolpath planning and optimization of the micro-milling process remain so underdeveloped at this time, with conflicting results and conclusions on its effect as reported above. Toolpath development must become an important area for future research to really advance the micro-milling process as a whole, particularly for freeform machining, so that it will find further application in industries such as optics and biomedical devices manufacturing.

### Cutting fluid

Cutting fluids are essential to all cutting processes. They can fall into categories of coolant, lubricant, or both. They are used extensively to supply a steady flow of fluid into the working area to cool, lubricate, flush away chips, reduce friction forces, etc. This leads to increased tool and machine tool life, improved surface quality, effective chip management, and more efficient machining. Other desirable properties of a cutting fluid are being nontoxic and safe to handle, while preventing any chemical corrosion or degradation of the tools or components [158]. Cutting fluids can be applied to the working area in various ways, such as by compressed air as minimum quantity mist, in a flooding process, and at high and low pressure. Fang et al. [159] even introduced chlorine mist and cooled air with success. Koklu and Basmaci [156] implemented flood coolant during micro-milling and it was shown that the tool marks were homogeneously formed, while the deterioration of the machined surface was minimized. However, this process of applying cutting fluid to the working area is inefficient, expensive, and can cause negative effects to operator’s health as well as the environment through bacteria and fungi growth, which leads to bad odor, dissociation of emulsion, reduction in lubrication, and spread of diseases [160].

In terms of reducing the required amount of cutting fluid, Li and Chou [161] analyzed the performance of the minimum quantity lubrication (MQL) technique, as depicted in Fig. 15b, in near micro-milling with respect to dry cutting on process outputs. It was found that the application of MQL substantially improved the tool life, surface roughness, and burr formation compared dry cutting based on slotting tests with micro end mills on a mesoscale machine tool. Huang et al. [162] used a nanofluid/ultrasonic atomization MQL technique with ultrasonic dispersion during micro-milling of SKD11 steel. They compared different MQL nanofluids in terms of effects on micro-milling cutting force, micro-milling temperature, micro-milling tool wear, and surface burr. Pham et al. [163] revealed that high-viscosity ionic lubricants provided a slightly better machined surface and exhibited extremely low volatility compared with conventional oils or other lubricants in the micro-milling process. Javaroni et al. [160] showed that the conventional cutting fluid provided better results for the output variables analyzed in advanced ceramics grinding compared to those of the MQL process. However, the MQL can still present satisfactory results considering the economic, health, and environmental benefits offered by this technique. MQL can also greatly reduce the consumption of cutting fluid, thereby reducing environmental pollution and associated costs due to lower volume requirement, subsequent post-processing, disposal, etc. In addition, MQL can also enhance the ability of cutting fluid to enter the cutting zone, which can greatly improve the cooling and lubrication effects. In general, MQL is a highly efficient and low-cost cutting fluid technique [164].

Micro-milling processes may not always require cutting fluid flooding, such as when light machining some polymers, ceramics, and alloys and when the risk of contamination specifically does not allow it, e.g., machining some biomaterials for biomedical implants. Therefore, dry cutting conditions of nonconventional approaches are necessary to lower the cutting temperature while ensuring efficient chip evacuation. Effective nonconventional methods include MQL as discussed, dry cutting, chilled air, cryogenic cooling, as well as the use of solid lubricants, all of which have been shown to be viable substitutes to cutting fluid while maintaining machining performance [165]. The application of cryogenic cooling and chilled air can actually lead to lower cutting forces, surface roughness, and tool wear during some machining processes owing to the reduction in the coefficient of friction at the interface of the tool and chip; however, the opposite can occur as well when the mechanical properties of some materials, such as microhardness, increase under the condition of being cryogenically cooled [166]. Dry machining has the benefits of reducing contamination and disposal, and it is the most environmentally safe option. However, dry machining causes problems of high temperature, high friction, oxidation, the inability to achieve close tolerances due to thermal expansion of the workpiece, as well as metallurgical damage to superficial layers [167]. All of these methods are heavily dependent on the material being machined, exact machining process, size of the chip to be formed, design and geometry of the tool, etc. However, most of the work carried out so far has been based on conventional machining processes, such as macro-milling and turning. Therefore, further investigations of the MQL process, dry machining, chilled air cutting fluid, and cryogenic cooling must be carried out specifically for micro-milling processes under dry cutting conditions, while maintaining efficient chip removal, low coefficient of friction, adequate cooling, and achieving close tolerances.

The environmental impact of cutting fluids is currently an important consideration in micro-milling; however, it is applicable across all machining processes. It will even become more important in the coming years as industries strive to become more environmentally and economically sustainable. This has led to interesting areas for future research in micro-milling. For example, Chen et al. [168] developed a novel electrochemical micromembrane technology to demulsify oily wastewater and recover oil from oil-in-water cutting fluids. Similarly, Shen et al. [169] presented an approach to recover cutting fluids and SiC from slurry waste. Although eco-friendly cutting fluids should be the target to move forward, the importance of maintaining the process outputs will remain. Burton et al. [170] conducted an investigation into effectively obtaining a vegetable oil-in-water emulsion through ultrasonic atomization. Their experimental results were very positive, showing lower cutting forces, smaller chip thickness, and less burr formation for the micro-milling process. Li et al. [164] further developed this vegetable oil-in-water emulsion-based cutting fluid by dispersing graphene and using the MQL technique, meeting the demands of cleaner and sustainable manufacturing. The reduction and recovery of cutting fluid can reduce both the cost and environmental damage caused by machining, which should be viewed as an integral component of every micro-milling manufacturing chain. However, although the environmental aspect is an important consideration, the focus of research on micro-milling cutting fluids should first consider the lubrication properties to minimize BUE, friction, burr formation, ploughing, etc., followed by coolant properties to regulate the temperature at the cutting zone.

## Advanced processes

Currently, the micro-milling process is limited by the inherent constraints of cutting material removal mechanisms at the microdomain, which include chip formation, size effect, and process stability. However, these constraints may be overcome by the application and combination of new technologies with the micro-milling process, such as micro-rotary ultrasonic vibration-assisted milling (μRUAM), laser-induced oxidation-assisted micro-milling (LOMM), and atmospheric-pressure plasma jet-assisted micro-milling.

### Micro-rotary ultrasonic-assisted machining

Micro-milling has been shown to be an advantageous machining process for manufacturing surfaces, features, and structures in the microdomain with high accuracy and precision. However, the application of micro-milling in the mold, optics, and biomedical industries requires that this process must be suitable for machining typical difficult-to-machine materials, from very hard and wear resistant metallic alloys to very brittle ceramics or deliquescent crystal materials. One of the major efforts directed toward processing these difficult-to-machine materials has been the application of μRUAM [158]. This process applies an ultrasonic frequency vibration in the range of 20–100 kHz with an amplitude between 5 μm and 50 μm at the tool tip in one or more directions, e.g., axially or radially. So far, μRUAM has been shown to reduce cutting forces and improve tool life during machining, owing to the working mechanisms of material removal, which form smaller chip sizes, reduce contact at the tool-workpiece interface, reduce frictional forces, and inhibit crack propagation on very brittle materials. However, strict control over the machining and vibration parameters, direction of vibration, and the process as a whole is necessary, as tool life can actually be diminished with wrong parameter selection [171, 172].

In relation to the abovementioned materials, Jin and Xie [173] presented an experimental study on the surface generation in μRUAM of a BK-7 optical glass using a 2-flute micro end mill tool. They showed that the vibration direction had a major effect on surface quality, with vibration applied in the normal direction improving the machined surface. The vibration assistance also enhanced the brittle-ductile transition of glass and therefore reduced the surface damage. Finally, they concluded that a higher vibration frequency improved the surface quality by reducing the surface waviness. Bian et al. [174] also conducted an experimental investigation on micro-milling of brittle materials, but on ZrO_2_ ceramics with diamond-coated micro end mills. It was found that the chips formed in the ductile mode were long and thin curled strips with a smooth back surface, leading to less edge and surface chipping. However, the compressive forces due to the ductile mode of material removal presented an increasing trend with random fluctuations of the cutting force, leading to higher tool wear.

**Fig. 13:**
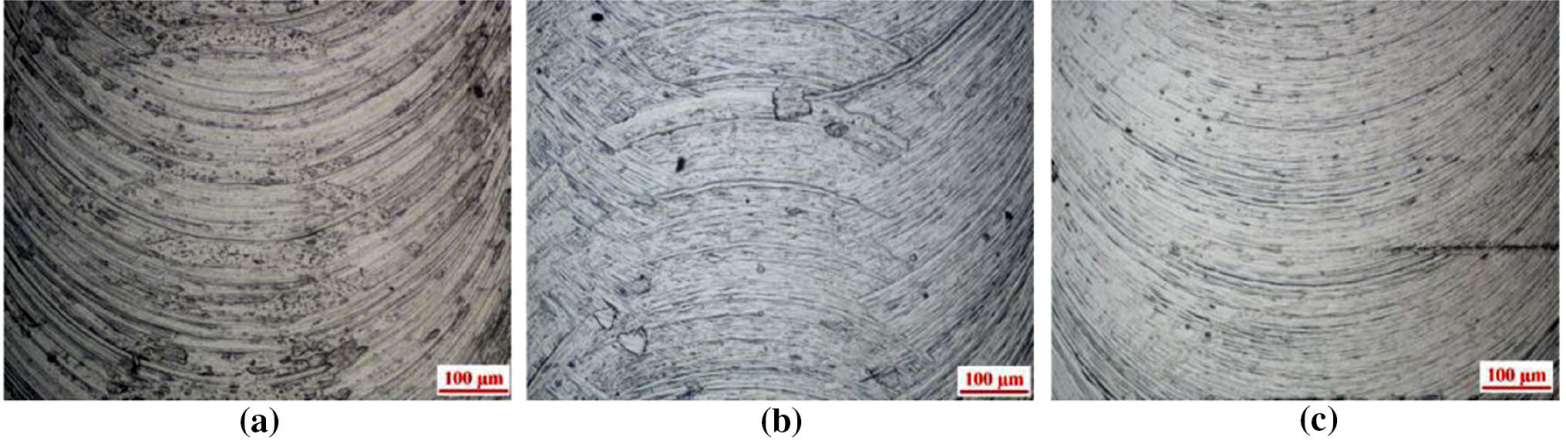
Machined surface after ultrasonic vibration micro-milling experiments at amplitudes of **a** 0 µm, **b** 2 µm, and **c** 4 µm, showing that ultrasonic vibration-assisted micro-milling can reduce surface defects and machining marks and thus improve the surface quality (Reprinted from “Machinablity improvement with ultrasonic vibration–assisted micro-milling” by Xu et al. [176], with permission from Sage)

With respect to very hard and wear resistant metallic alloys in particular, Li and Wang [175] recorded lower tool wear and better surface quality when the cutting speed was much less than the maximum vibration during μRUAM compared to conventional milling. The material tested was AISI H13, which was suitable for manufacturing molds and dies. Xu et al. [176] performed μRUAM research on titanium alloy TC4 and aluminum alloy 6061T6 with ultrasonic vibration in the radial direction. Their experimental results also verified that μRUAM could reduce the cutting force, while improving surface quality by reducing machining marks. Burr formation was also substantially lessened in μRUAM, compared to conventional milling, as shown in Fig. 13. Finally, the team determined that the size effect appeared at much lower feed rates than in conventional micro-milling. They proposed that vibration-assisted machining at the microdomain triggered a change in the material removal mechanism. According to the authors, “impulse impact accelerates the generation and propagation of tiny cracks in the workpiece material, which reduces the binding force inside the grains of the material.” Although this may be significant in reducing and eliminating the size effect in micro-milling, an in-depth analysis will be necessary for future work. Feng et al. [177] proposed a predictive model to estimate flank tool wear to a high accuracy. They found that a smaller axial depth of cut, larger feed per tooth, or higher cutting speed would result in higher flank wear rate, while the effects of the vibration parameters were less significant. Clearly, μRUAM is an interesting area in the development of micro-milling, as it may reduce some inherent limitations of the current conventional micro-milling process. Much work needs to be carried out concerning the physical process as well as process inputs, while research will now require focusing on more theoretical work rather than experimental work to fully characterize the process.

### Laser-induced oxidation-assisted micro-milling

**Fig. 14:**
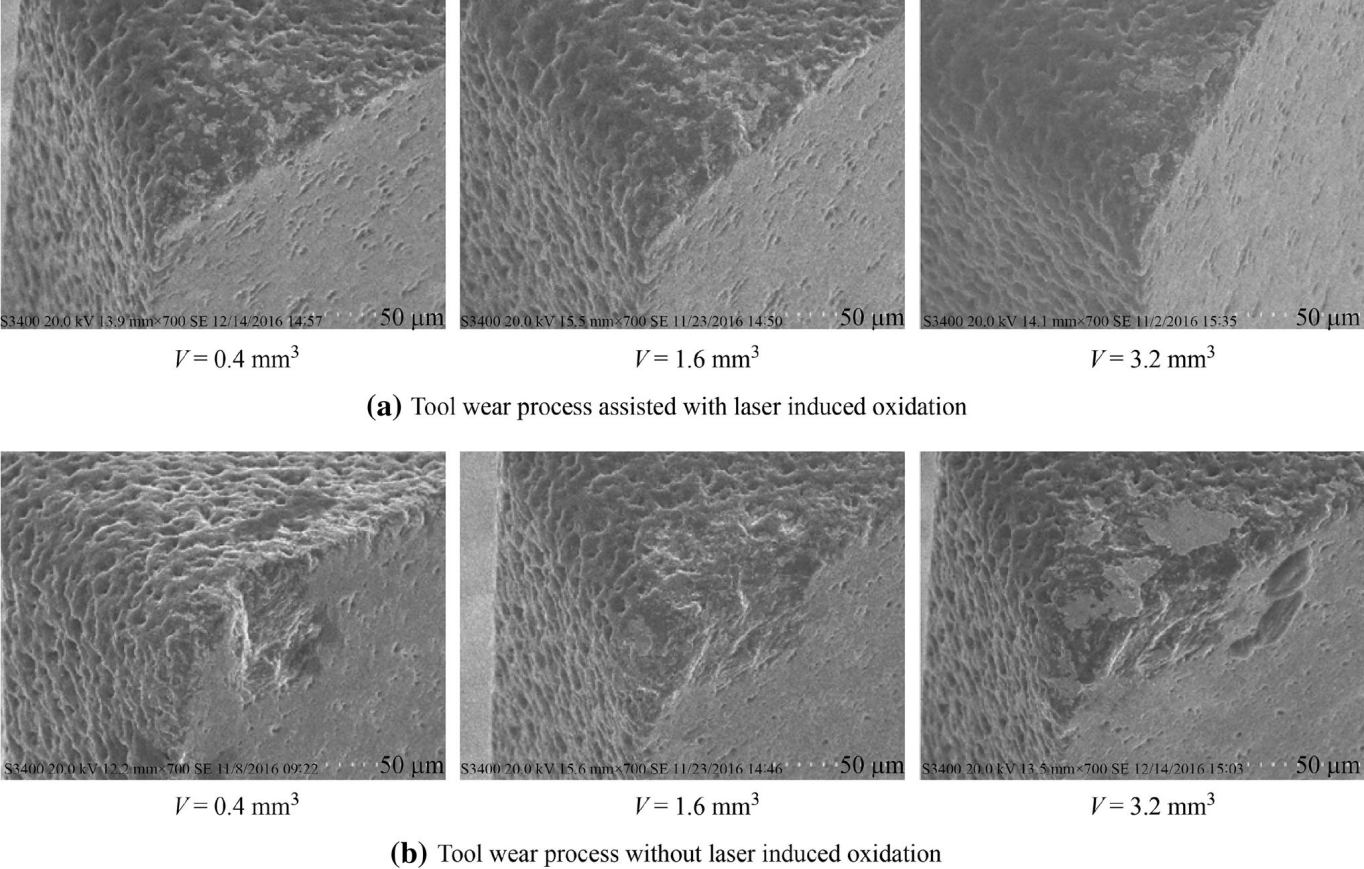
Tool wear process with different material removal volumes: **a** with laser-induced oxidation and **b** without laser-induced oxidation (Reprinted from “Laser-induced oxidation of cemented carbide during micro-milling” by Wu et al. [182], with permission from Elsevier)

LOMM is derived from laser-assisted micro-milling (LAMM). LAMM combines the mechanical process of micro-milling with highly localized thermal softening of the hard material by continuous wave laser irradiation. Subsequently, the softened material is removed by micro-milling [178]. Compared to the conventional micro-milling process, the cutting force in LAMM is substantially decreased and the tool life is prolonged. However, a high laser power is required to soften hard materials such as ceramics. This would result in the ablation of workpiece material, expansion of heat affected zone, and formation of microcracks [179]. Therefore, Yang et al. [179] proposed the novel process of LOMM, which used a relatively low-power laser to irradiate the surface of a ceramic material. An oxidation reaction between the ceramic material and oxygen occurs, forming a loose and porous oxide layer, which can be removed easily through the mechanical process of micro-milling with a low cutting force thereafter. Compared to the conventional micro-milling, the surface quality by LOMM was better, and the machining efficiency was improved by 104%. Xia et al. [180, 181] also presented a study on Ti-6Al-4V using this novel process. They showed that LOMM effectively decreased the cutting force and tool wear and prolonged the service life of the tool. They verified that the cutting force when removing the oxide layer in LOMM was 50%–65% lower than when removing the material in conventional micro-milling under the same cutting parameters. It was also noted that the top burr width of the machined microgroove and tool wear were smaller by LOMM. Wu et al. [182] also confirmed that far less tool wear occurred for LOMM compared to conventional micro-milling, as shown in Fig. 14. This is a very new and promising area of research for the micro-milling industry, with considerable opportunities for theoretical and experimental works yet to be presented.

### Atmospheric plasma jet-assisted micro-milling

**Fig. 15:**
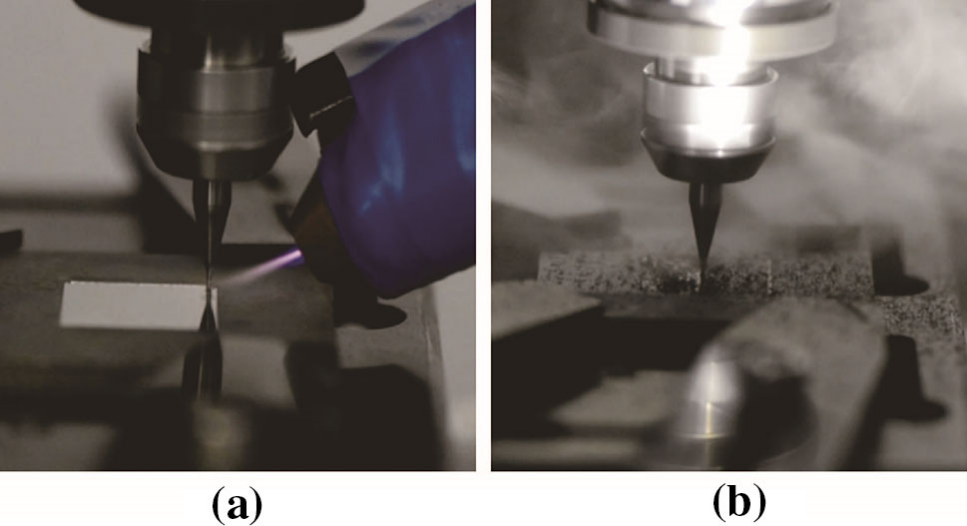
Processes of machining Inconel 718 alloy **a** plasma-assisted micro-milling process and **b** MQL process (Reprinted from “Atmospheric pressure plasma jet-assisted micro-milling of Inconel 718” by Mustafa et al. [184], with permission from Springer Nature)

Atmospheric-pressure plasma jet-assisted micro-milling is another underdeveloped assisted micro-milling process, which was proposed by Katahira et al. [183]. The team performed a feasibility study to investigate the effects of the application of an atmospheric-pressure plasma jet during PCD micro end milling, which compared machined SiC surfaces for both with and without the application of plasma jet. It was revealed that with the plasma jet, the formation of a high-quality surface was possible. Moreover, it was also highly effective in improving the chip formation process by imparting hydrophilicity to the tool and workpiece surfaces, as well as removing surface contamination at the tool edge during machining. However, no additional work had been presented on this process until Mustafa et al. [184] very recently. They also confirmed that atmospheric-pressure plasma jet-assisted micro-milling was a promising assisted technology with respect to the micro-milling process, as it provided the lowest surface roughness values among various cutting environments: dry, nitrogen jet, plasma jet, MQL, and plasma jet combined with MQL (see Fig. 15). The material tested was Inconel 718. It was determined that the plasma jet could promote fracture of the nickel surfaces and therefore reduce the cutting force. However, it was demonstrated that the residual stresses in micro-milled machined surfaces were compressive, and atmospheric-pressure plasma jet tended to increase such compressive residual stresses. Again, far more work needs to be carried out to begin characterizing this process, which has great potential for reducing the inherent issues of machining in the microdomain.

## Applications

The development of machine tools and the manufacturing technology as a whole has led to high-precision micro-milling processes in both research and industrial fields. The increasing demand for micro-structured parts and products with functional surfaces requires enhancing the process efficiency to develop new technologies and improve existing ones, so that a faster and more reliable production can be achieved [185]. The application of the micro-milling process ranges from fabrication of microstructures and micro-components to micro-texturing and mold manufacturing for industries such as electronics, aerospace, aeronautics, and biomedicine.

### Micro-structures

**Fig. 16:**
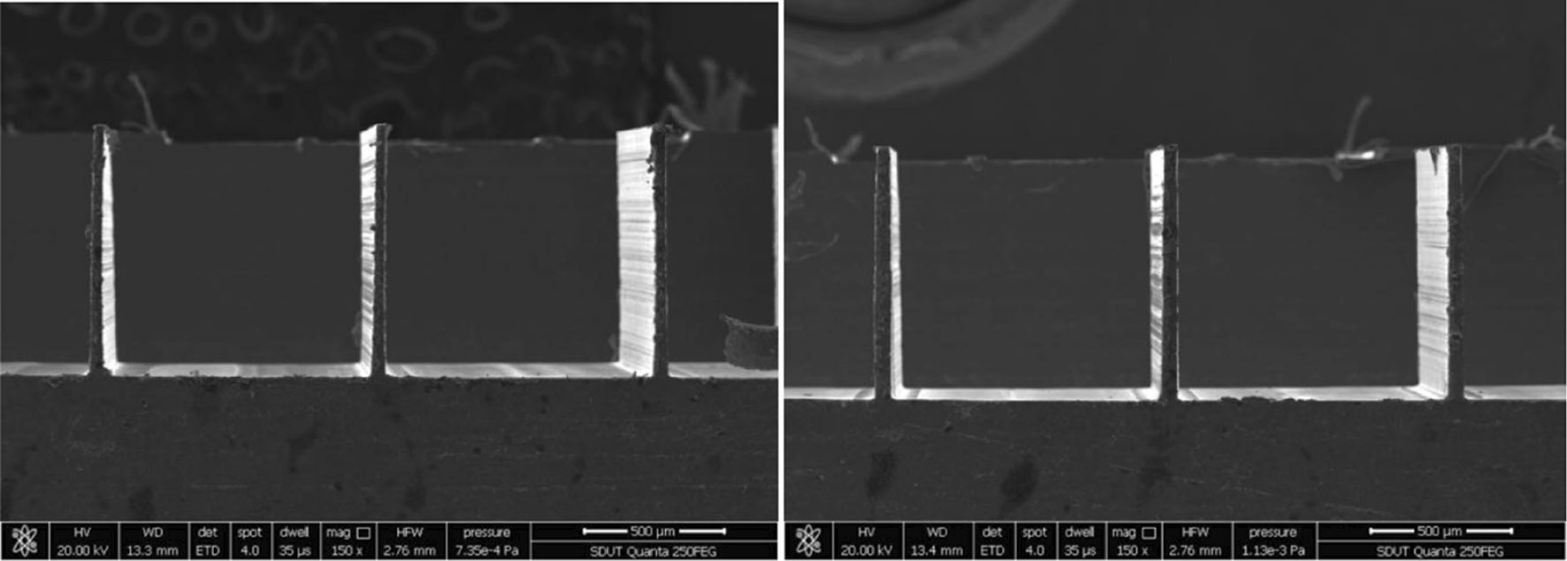
Thin-wall features of 900 um in height (Reprinted from “Experimental study on micro-milling of thin walls” by Wang et al. [201], with permission from IOP)

One of the earliest and most employed application areas for the micro-milling process is in microstructure and micropart fabrication. Microdevices can be defined as having at least two critical dimensions in the sub-millimeter range with at least one critical dimension significantly smaller than 0.1 mm and with tolerance ranges of a few microns to nanometers [84, 186, 187]. The functionality of components can be improved by surface modifications such as microstructures using the micro-milling process. These structures can cause changes in the mechanical properties of the components, as reported by Godart et al. [188], who determined that 50 µm wide microstructures with a depth of 10–20 µm could increase tensile strength and decrease the fracture elongation in commercially pure-titanium workpieces. A basic example of such structure is a micro-thin wall, which usually refers to a cantilever structure with a thickness below 100 μm and a height to thickness ratio greater than 10 [189]. Accurate and precise removal of material to form micro-thin wall structures is very difficult to accomplish in reality, especially for metallic alloys that tend to deform plastically when the wall thickness is in microns, as exhibited in Fig. 16. As the thickness of the wall is decreased, failure of the microstructure begins to occur owing to the wall thickness exceeding the material threshold of rigidity and strength. Other microstructures include pipes, blades of an impeller or turbine, walls of a microchannel, microcolumns, and fins of a heat exchanger. At present, these microstructures have been widely applied in micro-fuel cells [190], microfluidic chip channels [191], and EDM electrodes [192, 193].

In the energy and electronics industries, the enhancement in efficiency of heat transfer devices is crucial as the trend toward miniaturization of devices requires better understanding of heat transfer in small dimensions [194]. Repeated rib surfaces are known for their effectiveness in enhancing heat transfer and they are widely required in many scientific and industrial applications. Further improvements to these microstructures were made by Wang et al. through the introduction of a textured asymmetric arc rib structure on which microstructure arrays of secondary microgrooves were superimposed [195]. It was then verified by Zhao et al. [196] that these hierarchical microstructures, composed of a primary microstructure and secondary micro V-grooves, could be machined well by an ultra-precision micro-milling process using a one-step cutting operation and a diamond tool.

Another application of microstructures that can be produced by micro-milling is for mass sensing in microelectromechanical system (MEMS) devices [197], which are used in the telecoms market, e.g., mobile phones and optical modulators [198], using lithium niobate (LiNbO_3_) material. LiNbO_3_ is a crystalline material also often used in surface acoustic wave sensors and optical drives owing to its superior electrical, optical, and physical properties [199]. However, because of its low toughness, it is considered a difficult-to-machine material, which has conventionally been used as a substrate with deposited microstructures, rather than machined. However, owing to the ever-increasing demand for higher efficiency, direct fabrication of structures on the surface of LiNbO_3_ is now necessary, and this can be accomplished easily by micro-milling, according to Huo et al. [200]. Clearly, micro-milling is a direct and effective manufacturing method for fabricating microstructures with complex 3D shapes. Nevertheless, the limitations of material deflection, plastic deformation, and burr formation during machining of such microstructures remain to be major issues that need to be addressed.

### Micro-texturing

Another major application of the micro-milling process is in micro-texturing or micro-patterning to reduce frictional forces and reduce wear between parts in industries such as automotive and biomedicine. As summarized by Chen et al. [202], functional microtextured surfaces have high aspect ratio features, which enable the component to have superior properties such as reduced adhesion friction [203], improved lubricity [204], increased wear resistance [205], ability to manipulate hydrophilic performance [206], as well as influence optical properties [207]. Three possible mechanisms by which surface micro-texturing improves tribological performance, as outlined by Chen et al. [208], are described as follows. Firstly, the textured surface can increase the load-carrying capacity by serving as micro-hydrodynamic bearings for hydrodynamic lubrication [209]. Secondly, surface micro-textures can act as a second lubricant source to permeate the surface and reduce friction and wear between both surfaces, creating a lubrication boundary [210]. Finally, surface micro-textures can reduce the ploughing induced by abrasive wear and deformation between components by capturing wear debris between the texture features [211]. It was also shown by Kovalchenko et al. [212] that arrayed dimples on contact surfaces under lubrication helped to establish hydrodynamic pressure and decrease the friction force. These three mechanisms of tribological performance improvements have significant potential in orthopedic implants for arthroplasty procedures related to replacement of joints, such as hips, knees, or elbows. It has already been shown that micro-milling is a high-precision machining process suitable for difficult-to-machine materials, such as titanium alloys, cobalt-chrome alloys, and ceramics, all of which are commonly used for orthopedic implants.

**Fig. 17:**
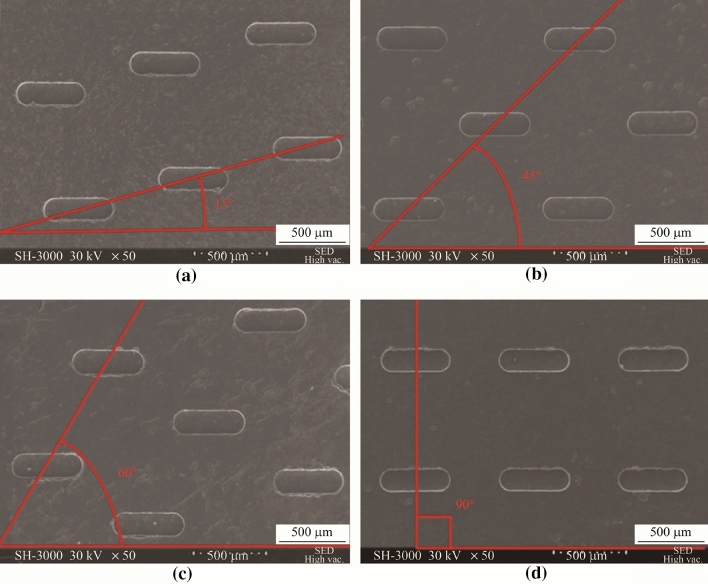
Microtexture SEM profiles with different distribution angles: **a** *θ* = 15°, **b** *θ* = 45°, **c** *θ* = 60°, and **d** *θ* = 90° (Reprinted from “Effects of micro-milled malposed dimple structures on tribological behavior of Al-Si alloy under droplet lubricant condition” by Chen et al. [208], with permission from Springer Nature)

Micro-textures can be fabricated by employing various techniques, including laser machining [213] and etching [214]. However, the most cost effective and efficient method will always be rapid and direct machining owing to the relatively low power consumption, precise and accurate 5-axis CNC programming, and high surface finish, all of which are characteristics of the micro-milling process. Although the application of micro-milling to fit this purpose is relatively new, there has been some interesting works presented lately. For example, Syahputra and Ko [215] developed a rapid process for acquiring complex texture data by using an image processing technique for the micro-milling process in which a complex surface texture representing human skin is transferred to a metal surface. Similarly, Chen et al. [208] concluded that the friction performance of micro-milled Al-Si alloy ZL10 surfaces could be enhanced by malposed rectangle dimple micro-textures, as illustrated in Fig. 17. Their results showed that the average friction coefficient was reduced by 6.93%, while optical micrographs indicated that the microtextured specimens exhibited the narrowest and shallowest wear track, in comparison to untextured specimens. Micro-milling using a ball nose end tool is another viable technique for creating such micro-textures, as demonstrated by Graham et al. [216]. By tilting the tool at an inclined angle, the spindle speed and feed rate can be adjusted so that the flutes of the cutter create periodic patterns in a workpiece surface. However, the problem of burr formation associated with the machining of microgrooves and micropatterns remains an important issue. In an attempt to solve this problem, Fang et al. [217] studied the effects caused by cutting parameters, work material, and cutting methodology. However, more detailed work into tool geometry will be necessary to limit burr formation during micro-milling. Evidently, micro-milling is an efficient and versatile manufacturing technique, ideal for rapid machining of micro-textures and micropatterns across a broad range of materials and industries. However, the limited research available at this time indicates a significant research gap, which must be filled so that this precision machining process can be applied in the field of orthopedic implant manufacturing.

### Mold making

**Fig. 18:**
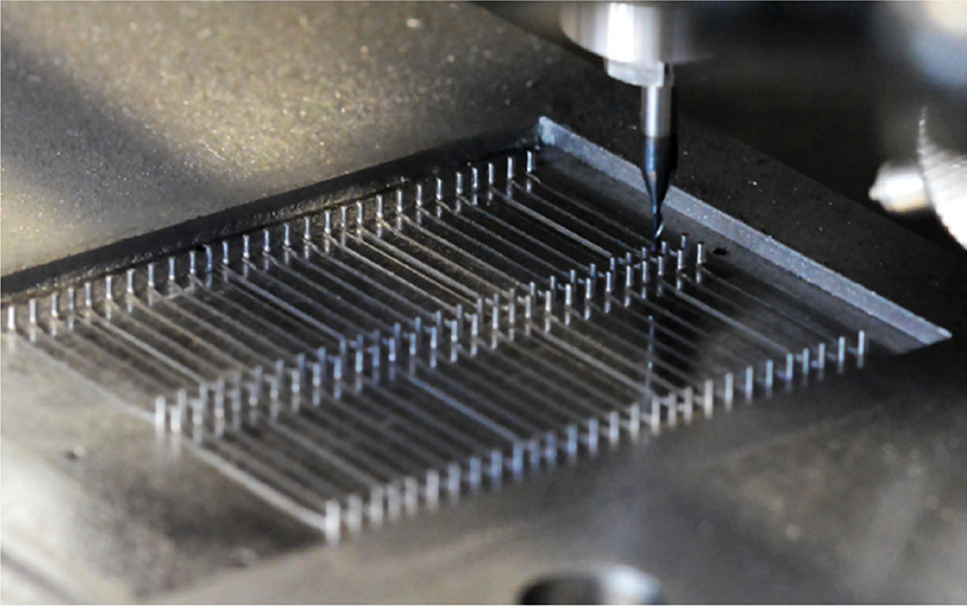
Micro-milling of a lab-on-a-chip microfluidic mold: 4 arrays of 28 pins with 0.8 mm diameter and 2 mm height (Reprinted from “Impact of deep cores surface topography generated by micro-milling on the demolding force in microinjection molding” by Masato et al. [225], with permission from Elsevier)

The most significant application of the micro-milling process is in the mold making industry, because it permits precise, rapid, and accurate machining of high aspect ratio microfeatures, such as microchannels, microarrays, and thin walls, as already discussed. These molds are essential to microinjection molding, micro-hot embossing, and nanolithography industries for the polymer micro/nano mass replication of features and surfaces [218]. The most obvious contribution is rapid manufacturing and prototyping of molds and mold inserts through finishing processes of roughed out mold cavities, providing a quick and efficient processing time. A distinctive application example of this is for the microfluidics industry, which is important for the employment of disposable medical sensors. This highly significant area for micro-milling application in the microfluidics industry is in functionally optimized surfaces through patterned microstructures on miniaturized bioreactor components, also known as “lab-on-a-chip”, as shown in Fig. 18. The microfluidic chip provides a cheap and disposable platform for production and testing of pharmaceutical and biomedical products [219]. The possibilities for such microfeatures may also offer an important role in distinguishing biofilm behavior in the future [220]. The miniaturized size also allows for lower power consumption and greater portability, while utilizing smaller volumes of reagents and samples, which are extremely important to the microfluidics industry [221]. From a manufacturing perspective, the micro-milling process allows for rapid prototyping of microfluidic devices [222].

However, several challenges remain in micro-milling of molds and dies, namely burr formation and thin-wall deformation. Thus, it is important to identify and investigate these issues in applying this process in the mold making industry. Saptaji determined that micro-milling is capable of creating the features necessary for a thin microfluidic embossing mold, with a thickness and feature height of approximately 160 μm and 100 μm, respectively [223]. The appropriate selection of the micro-milling strategy is also crucial in achieving designed micro-lens array surfaces, according to Gao et al. [224], while different machining strategies have different machining surface textures due to the cutting direction. An important work by Ardila et al. [185] aimed at improving the entire micro-milling production chain to generate knowledge about related process stages, including potential improvements of productivity and quality. The team concluded that to increase the application of micro-milling in the mold industry, the micro-milling process must satisfy the productivity and quality standards, confirming that the process needs further research to comply with these requirements.

## Conclusions and perspective

The high-precision micro-milling process was shown to be a very effective and versatile manufacturing process, capable of machining a broad range of difficult-to-machine materials for applications that require tight tolerance, good surface integrity, and efficient machining across all industries. Although micro-milling is not a particularly new manufacturing process and has been the focus of considerable research work throughout the years, new and novel applications for this process are constantly being established in the industry. The flexible and versatile nature of micro-milling has guided this precision process from direct manufacturing of MEMS components to 5-axis CNC machining of precision molds for micro/nano-polymer replication. Currently, further development of the process is being driven by the necessity for rapid and highly accurate micro-patterning and micro-texturing of surfaces, for producing large numbers and arrays of micro-sized features such as dimples and rectangular pockets to increase lubricity and reduce friction forces between wear parts. The driving force behind this latest development is the requirement of the bio-implant industry to produce such features in a precise and efficient manner and to improve the tribological performance of orthopedic implants, thereby extending implant life. Therefore, the development of efficient and precise micro-milling to produce microstructures, micro-textures, and high-quality molds has promoted a sustainable future for the micro-milling process, with interesting new areas and applications to overcome current limitations of other technologies.

However, as mentioned throughout the review, many problematic and inherent issues of the micro-milling process prevent its application in industries until further research and investigations are carried out. Such issues include the phenomena of downscaling machining to the microdomain, i.e., the size effect, chip formation mechanisms, and fundamental process instabilities involved. Primarily, burr formation during channel or slot milling is the most critical issue limiting the use of micro-milling in microfluidic chip molds, where undesirable projections of the material form as a result of the plastic flow from cutting and shearing operations. Since post processes such as deburring are costly and are non-value added operations, understanding and control of burr formation are research topics with high relevance to industrial applications, with much work yet to be carried out. Similarly, adhered materials on the tool cutting edge, i.e., BUEs, have a large influence on the machining process outputs. The effect is particularly significant with the reduction in diameter of microtools, where even small material deposits can lead to substantial geometric errors and poor surface finish. Accordingly, more thorough investigations into BUE prevention, while looking toward stable BUE development, will have profound impacts on the current state of the micro-milling process. Likewise, reduction and altogether elimination of process instabilities, such as tool deflection, tool runout, and chatter, will advance the development of micro-milling in the future. Reduction of such instabilities will make micro-milling evolve into a high-precision process, i.e., with reduced feature size from a few microns down to the submicron level. This will also significantly contribute to decreasing the cutting force, surface roughness, cutting temperature, and tool wear.

High tool wear is another major issue currently impeding the micro-milling process, leading to less efficient machining, poorer surface finish, and higher costs. Further examination of the multifactor effect of the process inputs involved in micro-milling offers another significant area for micro-milling research, specifically in relation to tool optimization, e.g., tool geometry, material, microstructure, and coating. Furthermore, the effects of workpiece material microstructure, CNC toolpath, and cutting fluid, will all carry an important role for future work. Similarly, combined processes of new technologies with the conventional micro-milling process offer suitable solutions to overcome inherent constraints of cutting material removal mechanisms at the microdomain. Finally, current and future trends of the micro-milling process are discussed in detail in relation to its industrial applications, while important insights into further development are considered and significant research gaps are identified.
